# Glucose limitation activates AMPK coupled SENP1-Sirt3 signalling in mitochondria for T cell memory development

**DOI:** 10.1038/s41467-021-24619-2

**Published:** 2021-07-16

**Authors:** Jianli He, Xun Shangguan, Wei Zhou, Ying Cao, Quan Zheng, Jun Tu, Gaolei Hu, Zi Liang, Cen Jiang, Liufu Deng, Shengdian Wang, Wen Yang, Yong Zuo, Jiao Ma, Rong Cai, Yalan Chen, Qiuju Fan, Baijun Dong, Wei Xue, Hongsheng Tan, Yitao Qi, Jianmin Gu, Bing Su, Y. Eugene Chin, Guoqiang Chen, Qi Wang, Tianshi Wang, Jinke Cheng

**Affiliations:** 1 State Key Laboratory of Oncogenes and Related Genes, Renji Hospital Affiliated; Shanghai Key Laboratory for Tumor Microenvironment and Inflammation, Department of Biochemistry and Molecular Cell Biology, Shanghai Jiao Tong University School of Medicine, Shanghai, China; 2 Department of Urology, Renji Hospital Affiliated, Shanghai Jiao Tong University School of Medicine, Shanghai, China; 3 Department of Urology, Xinhua Hospital Affiliated to Shanghai Jiao Tong University School of Medicine, Shanghai, China; 4 Institute of Biophysics, Chinese Academy of Sciences, Beijing, China; 5 Clinical Research Center, Shanghai Jiao Tong University School of Medicine, Shanghai, China; 6 College of Life Sciences, Shaanxi Normal University, Xi’an, Shaanxi China; 7 Department of Thoracic Surgery, Zhongshan Hospital, Fudan University, Shanghai, China; 8 Shanghai Institute of Immunology, Shanghai Jiao Tong University School of Medicine, Shanghai, China; 9 Institutes of Biology and Medical Sciences, Soochow University Medical College, Suzhou, Jiangsu China

**Keywords:** Nutrient signalling, T cells, Sumoylation, Metabolomics

## Abstract

Metabolic programming and mitochondrial dynamics along with T cell differentiation affect T cell fate and memory development; however, how to control metabolic reprogramming and mitochondrial dynamics in T cell memory development is unclear. Here, we provide evidence that the SUMO protease SENP1 promotes T cell memory development via Sirt3 deSUMOylation. SENP1-Sirt3 signalling augments the deacetylase activity of Sirt3, promoting both OXPHOS and mitochondrial fusion. Mechanistically, SENP1 activates Sirt3 deacetylase activity in T cell mitochondria, leading to reduction of the acetylation of mitochondrial metalloprotease YME1L1. Consequently, deacetylation of YME1L1 suppresses its activity on OPA1 cleavage to facilitate mitochondrial fusion, which results in T cell survival and promotes T cell memory development. We also show that the glycolytic intermediate fructose-1,6-bisphosphate (FBP) as a negative regulator suppresses AMPK-mediated activation of the SENP1-Sirt3 axis and reduces memory development. Moreover, glucose limitation reduces FBP production and activates AMPK during T cell memory development. These data show that glucose limitation activates AMPK and the subsequent SENP1-Sirt3 signalling for T cell memory development.

## Abstract (web summary)

Memory T cells are particularly reliant on fatty acid oxidation as a source of energy. Here the authors show this reliance is controlled by AMPK sensing of glucose deprivation that triggers SENP1-Sirt3 signalling, driving fatty acid oxidation and memory differentiation in T cells via deacetylation of YME1L1 to induce mitochondrial fusion.

## Introduction

T cells are important contributors to adaptive immunity. Upon stimulation by antigen, T cells can differentiate into effectors and subsequently form memory cells. In effectors, some subsets, such as memory precursor effector cells (MPECs), differentiate into long-lived memory T cells and others, such as short-lived effector cells (SLECs), undergo apoptosis after antigen clearance^1,2^. Evidence indicates that the treatment of tumours using long-lived T cells can improve anti-tumour responses^2–4^, which is similar to results obtained with the use of vaccines against virus infection^5^.

T cell differentiation status is related to metabolic programming^6–8^. CD8^+^ effector T (T_E_) cells rely on aerobic glycolysis and oxidative phosphorylation (OXPHOS) to support their proliferation^9–11^, whereas memory T (T_M_) cells require fatty acid oxidation (FAO) for their survival^12–15^. Nutrient transport and utilization can affect T cell differentiation fate as well as function^16^. In the metabolic microenvironment, oxygen, nutrients and growth factors are limiting, along with immune reaction of effector T cells^16^. Previous studies have reported that these limiting factors can damage T cells leading to a gradual loss of their effector function and proliferation capacity^17^. These conditions may trigger T cells to reprogram their metabolism to ensure proper survival and/or effector function^16,18^.

In addition to metabolic programming, mitochondrial dynamics have been linked to T cell fate^7^. T_E_ cells have punctate mitochondria, whereas T_M_ cells maintain a fused mitochondrial network. As demonstrated using a genetic approach, mitochondrial fusion can facilitate efficient mitochondrial electron transport chain (ETC) activity and FAO in T_M_ cells, but elevates aerobic glycolysis in activated T cells^7,15^. Moreover, mitochondrial fusion can enhance T_M_ cell survival and activity^7^. Mitochondrial fusion is controlled by mitofusin-1 and mitofusin-2 (Mfn1/Mfn2) in the outer membrane and OPA1 in the inner membrane^19–21^. Interestingly, OPA1-mediated inner membrane fusion but not Mfn1- or Mfn2-mediated fusion is essential for T_M_ cell survival^7^. OPA1 increases cristae formation to support assembly of the respiratory chain supercomplex and OXPHOS in T_M_ cells^7^. However, regulation of OPA1-mediated mitochondrial fusion in T cell memory development is unclear.

AMPK is a critical regulator of T cell metabolism^22^ and functions as a metabolic checkpoint in effector cells to suppress mTOR activity in nutrient-limited environments^23^. Compared with effector cells, memory T cells have higher AMPK activity^14,23^. Furthermore, AMPK activation increases FAO and promotes memory development^14^. However, the physiological upstream signals of AMPK activation in T cell memory development are unclear.

Mitochondrial acetylation is a crucial regulatory mechanism for the activation of mitochondrial enzymes^24,25^. Sirt3, as a major mitochondrial NAD^+^-dependent deacetylase, is required for mitochondrial metabolic adaptation in response to various stresses. *Sirt3*-knockout mice have markedly increased acetylation levels of mitochondrial proteins in a variety of tissues^26,27^. Sirt3 can target a broad range of mitochondrial proteins^26^ and regulates mitochondrial complex I and complex II to activate electron transport in the mitochondrial inner membrane^28^ and promotes lipid biosynthesis via acetyl-CoA synthase 2 (AceCS2). Sirt3 also enhances FAO via long-chain acyl-CoA dehydrogenase (LCAD) under calorie-restricted conditions^29–31^. Moreover, Sirt3 can trigger global reprogramming of the mitochondrial protein acetylome under calorie-restricted conditions^26,31,32^. In addition to calorie restriction, other cellular stresses modulate Sirt3 function in mitochondrial metabolism^32,33^. In addition, Sirt3 can decrease the reactive oxygen species (ROS) level in mitochondria, indicating that Sirt3 activation is associated with various physiological or pathological processes, such as aging, hearing loss and tumorigenesis^34–36^. However, the function of Sirt3 in T cells is unexplored.

Mitochondrial Sirt3 has been identified as a SUMOylated protein^31^. SUMOylation suppresses Sirt3 deacetylase activity and then affects mitochondrial metabolism^31,32^. In response to calorie restriction, Sentrin-specific protease 1 (SENP1) deSUMOylates Sirt3 in mitochondria^31^ and SENP1-Sirt3 signalling promotes FAO and oxidative phosphorylation in cells and mice^31,32^.This study uncovers that the SENP1-Sirt3 axis is essential for T cell survival and memory development. SENP1-Sirt3 axis promotes FAO-fuelled OXPHOS and reduces YME1L1-mediated OPA1 cleavage in T cell memory development and T-cell-mediated anti-tumour immunity. Notably, low glucose activates the SENP1-Sirt3 axis via non-classical AMPK signalling in T cells, which promotes T cell memory development. Importantly, fructose-1,6-bisphosphate (FBP) as a glycolytic intermediate suppresses the AMPK-SENP1-Sirt3 signalling in memory development. Therefore, this study reveals that glucose limitation promotes AMPK-SENP1-Sirt3 axis via reducing FBP production, leading to enhanced T cell memory development.

## Results

### SENP1-Sirt3 axis is activated during T cell memory development

**Fig. 1:**
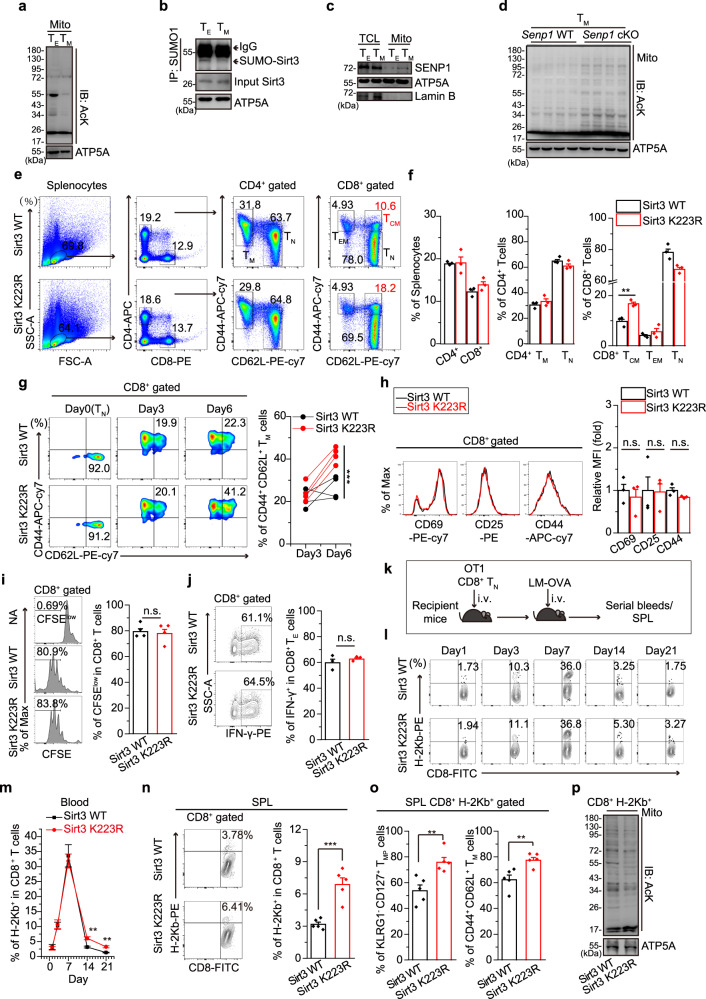
Activation of SENP1-Sirt3 axis promotes T cell memory development. **a** The global acetylation of mitochondrial proteins in CD8^+^ effector T (T_E_) and memory T (T_M_) cells (*n* = 4 biologically independent samples). **b** Sirt3 SUMOylation of T_E_ and T_M_ cells. **c** SENP1 protein in total cell lysates (TCLs) and mitochondrial lysates (mito) of T_E_ and T_M_ cells. **d** The global acetylation of mitochondrial lysates from *Senp1* WT and cKO CD8^+^ T_M_ cells (*n* = 4 biologically independent samples). **e**, **f** The T_M_ and naïve T (T_N_) cells of CD4^+^ T cell and the central memory T (T_CM_), effector-memory T (T_EM_), and T_N_ cells of CD8^+^ T cell in spleen were analysed (*n* = 3 mice, **P = 0.0059). **g**–**h** OT1 T_N_ cells were activated with OVA_257–264_ peptide + IL-2 for 3–6 days. The population of T_M_ cells were analysed (*n* = 4 mice, ***P = 0.0004) (**g**). The indicated surface markers were analysed on day 3 (*n* = 3 mice) (**h**). **i** The percentages of CFSE^low^ from activated OT1 T_N_ cells (*n* = 4 mice), nonactivation (NA). **j** The ratios of IFN-γ^+^ cells in activated OT1 T_N_ cells (*n* = 3 mice). **k**–**p** OT1 T_N_ cells were adoptive transferred into congenic recipient mice, and then the recipients were injected with LM-OVA (*n* = 5 mice) (**k**). The percentage of H-2Kb^+^ CD8^+^ T cells in the blood of recipients (**P = 0.0017 on day 14 and **P = 0.0037 on day 21) (**l**, **m**). The percentage of H-2Kb^+^ in the spleen CD8^+^ T cells of recipients on day 21 (***P = 0.00048) (**n**). The population of memory precursor (T_MP_) and T_M_ cells in the splenic CD8^+^ H-2Kb^+^ cells of recipients on day 21 (**P = 0.0055 in T_MP_, and **P = 0.0076 in T_M_) (**o**). The global acetylation of mitochondrial proteins in the splenic CD8^+^ H-2Kb^+^ cells from recipients (**p**). All immunoblots are representative of three independent experiments (**b**, **c**, **p**). Two-tailed unpaired *t*-test (**f**, **n**, **o**). Two-way ANOVA followed by Fisher’s LSD test (**g**, **m**). All data are shown as the means ± SEMs. n.s. not significant.

Cellular metabolic programme is tightly associated with the mitochondrial protein acetylation^29,33,37,38^. Since effector T cells (T_E_) and memory T cells (T_M_) perform different metabolic programmes^12–15^, we reasoned that the mitochondrial acetylation pattern in memory T cells would be different from that in effectors. Indeed, we detected remarkable lower mitochondrial acetylation levels in CD8^+^ T_M_ cells than that in CD8^+^ T_E_ cells (Fig. 1a and Supplementary Fig. 1a). Sirt3 as a major deacetylase in mitochondria plays a crucial role in regulating mitochondrial protein acetylation, and SUMO-specific protease SENP1 is a crucial regulator of Sirt3 activation via de-SUMOylation^31,32^. We thus speculated that the SENP1-Sirt3 axis may engage in T cell memory development. As expected, we detected the lower level of Sirt3 SUMOylation and more mitochondrial SENP1 proteins in T_M_ mitochondria than that in T_E_ mitochondria (Fig. 1b, c), suggesting that SENP1-Sirt3 signalling is activated along with T cell memory development. We further confirmed the activation of SENP1-Sirt3 axis by showing the higher mitochondrial acetylation level in T_M_ cells with *Senp1* conditional knockout (*Senp1* cKO) than that in *Senp1* wild-type (*Senp1* WT) T_M_ cells (Fig. 1d and Supplementary Fig. 1b). These data reveal that the SENP1-Sirt3 axis is activated in T cell memory development.

### SENP1-Sirt3 axis promotes T cell memory development

We next assessed whether the SENP1-Sirt3 axis affects T cell memory development. Sirt3 K223R, a SUMOylation mutant, has been reported to mimic the activation of the SENP1-Sirt3 axis^31^. A FACS analysis showed no difference between Sirt3 WT and Sirt3 K223R mice in the thymus T cell development (Supplementary Fig. 1c, d). However, we found that the central memory T cells (T_CM_, CD8^+^ CD44^+^ CD62L^+^) but not naïve (T_N_, CD8^+^ CD44^-^ CD62L^+^) or effector-memory T cells (T_EM_, CD8^+^ CD44^+^ CD62L^-^) were more abundant in the Sirt3 K223R spleen than that in the Sirt3 WT spleen under unchallenged condition (Fig. 1e, f). Also, the increased memory T cells in Sirt3 K223R spleen under unchallenged condition are ‘true’ memory T cells (T_M_, CD8^+^ CD44^+^ CD49d^+^) but not virtual memory cells (T_VM_) (CD8^+^ CD44^+^ CD49d^-^)^39,40^ (Supplementary Fig. 1e, f), and Sirt3 K223R promoted the differentiation of Treg cells in vitro other than CD4^ + ^helper T cells (Supplementary Fig. 1g). We further used Sirt3 K223R-OT1 mouse model to determine the role of SENP1-Sirt3 axis in T cell memory development. OT1 CD8^+^ naïve spleen T cells were cultured in vitro and activated by OVA_257-264_ plus IL-2. We detected that the memory population (CD8^+^ CD44^+^ CD62L^+^) in Sirt3 K223R OT1 mice was significantly higher than that in Sirt3 WT mice in 6 days after OVA_257-264_ treatment (Fig. 1g). However, there were no significant changes between Sirt3 K223R and Sirt3 WT in the expression of T cell early activation markers (CD69, CD25 and CD44), T cell proliferation (CFSE assay) and IFN-γ production in OT1 CD8^+^ T cells treated by OVA_257–264_ for 3 days (Fig. 1h–j). These data suggest SENP1-Sirt3 axis predominantly engaged in T cell memory development but not in effector T cells in response to antigen stimulation. Moreover, adoptive transferred mice were infected with *Listeria monocytogenes* to confirm T cell memory development in vivo. CD8^+^ naïve T cells from Sirt3 WT-OT1 and Sirt3 K223R-OT1 (donor cells) were adoptively transferred into congenic recipient mice, and then the recipients were injected (i.v.) with *Listeria monocytogenes* expressing-OVA (LM-OVA)^14,41^ (Fig. 1k). By serially bleeding and the detection of antigen-specific CD8^+^ T cells (OT1 tetramer^+^, H-2Kb^+^) in the blood of recipients, we found that both Sirt3 WT-OT1 and Sirt3 K223R-OT1 T_N_ cells mounted strong effector responses (day 3–7) (Fig. 1k–m and Supplementary Fig. 1h). However, the percentage of H-2Kb^+^ cells from Sirt3 K223R-OT1 donors was higher than that from Sirt3 WT-OT1 donors at day 14 to 21 after infection (Fig. 1k–m and Supplementary Fig. 1h). H-2Kb^+^ cells in the spleen of Sirt3 K223R-OT1 recipients were also more than those in Sirt3 WT-OT1 at day 21 post-infection (Fig. 1n and Supplementary Fig. 1i), indicating that Sirt3 K223R could promote T memory development. We further showed more T memory precursor cells (T_MP_, KLRG1^-^CD127^+^) and T memory cells (T_M_, CD44^+^CD62L^+)^ in the CD8^+^ H-2Kb^+^ splenocytes of Sirt3 K223R-OT1 recipients as compared to that of Sirt3 WT-OT1 recipients at day 21 after LM-OVA infection (Fig. 1o and Supplementary Fig. 1i). Moreover, we also detected lower mitochondrial acetylation in Sirt3 K223R CD8^+^ H-2Kb^+^ cells as compared to that in Sirt3 WT CD8^+^ H-2Kb^+^ cells (Fig. 1p). These results reveal that Sirt3 K223R can promote T cell memory development in vivo.

### Glucose limitation activates SENP1-Sirt3 axis via AMPK in T cell memory T cells

**Fig. 2:**
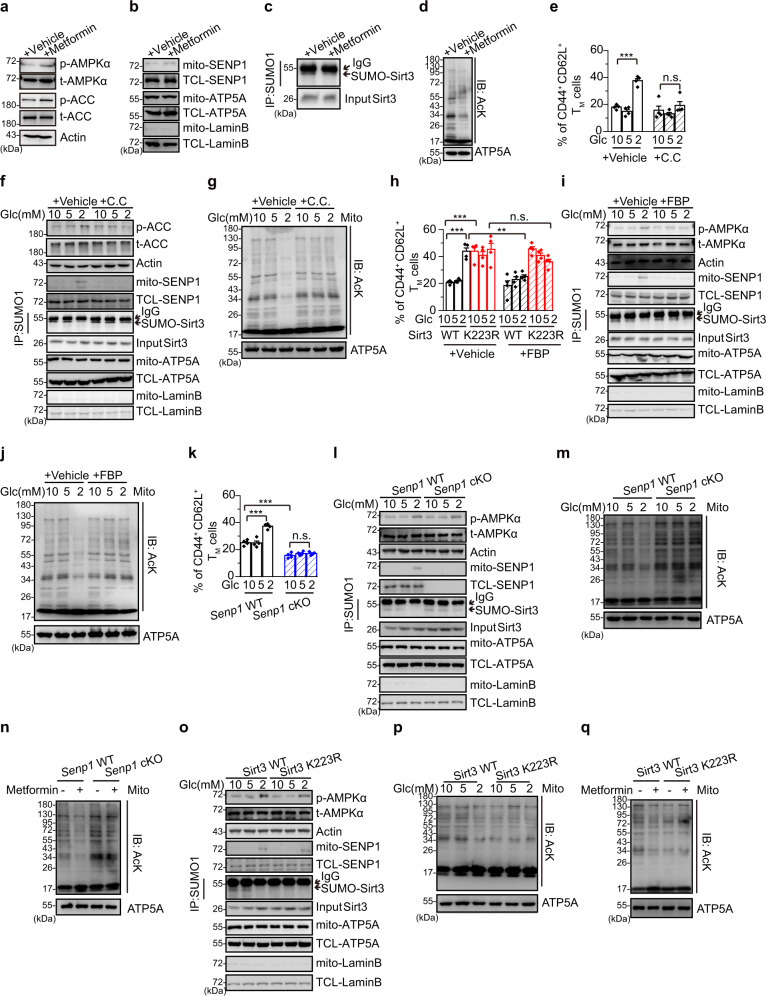
AMPK-SENP1-Sirt3 axis in T cell memory development. **a**–**d** The AMPK/ACC phosphorylation (**a**), mitochondrial SENP1 (**b**), Sirt3 SUMOylation (**c**), and the mitochondrial acetylation level (**d**) in activated T_N_ cells treated with metformin (2 mM, 3 days). **e**–**g** Activated T_N_ cells cultured with glucose (Glc, 10, 5 and 2 mM) and compound C (C.C, 10 μM, 3 days). The populations of T_M_ cells (*n* = 4 biologically independent samples, ***P < 0.0001) (**e**), the AMPK phosphorylation, mitochondrial SENP1, Sirt3 SUMOylation and the mitochondrial acetylation level were detected (**f**, **g**). **h**–**j** Activated T_N_ cells cultured with Glc (10, 5 and 2 mM) and fructose-1,6-bisphosphate (FBP, 5 mM, 3 days). The populations of T_M_ cells (*n* = 4 biologically independent samples, ***P < 0.0001 10 vs 2 mM Glc culture in vehicle treated WT cells, ***P < 0.0001 Sirt3 WT vs K223R cells in 10 mM Glc and vehicle, **P = 0.003 vehicle vs FBP treatment in 2 mM Glc cultured WT cells) (**h**), the AMPK phosphorylation, mitochondrial SENP1, Sirt3 SUMOylation and the mitochondrial acetylation level were detected (**i**, **j**). **k**–**m** Activated T_N_ cells cultured with Glc (10, 5 and 2 mM). The populations of T_M_ cells (*n* = 4 biologically independent samples, ***P < 0.0001 10 vs 2 mM Glc culture in WT cells, ***P = 0.0003 *Senp1* WT vs cKO cells in 10 mM Glc culture) (**k**), the AMPK phosphorylation, mitochondrial SENP1, Sirt3 SUMOylation and the mitochondrial acetylation level were detected (**l**, **m**). **n** The mitochondrial acetylation level of activated *Senp1* WT and cKO T_N_ cells with vehicle or metformin (2 mM, 3 days). **o**, **p** Activated Sirt3 WT and K223R OT1 T_N_ cells cultured with Glc (10, 5 and 2 mM). The AMPK phosphorylation, mitochondrial SENP1, Sirt3 SUMOylation and the mitochondrial acetylation level were detected. **q** Activated Sirt3 WT and K223R OT1 T_N_ cells cultured with vehicle or metformin (2 mM, 3 days). The mitochondrial acetylation level was detected. All immunoblots are representative of three independent experiments (**a**–**d**, **f**, **g**, **i**, **j**, **l**–**q**). Two-way ANOVA followed by Fisher’s LSD test (**e**, **h**, **k**). All data are shown as the means ±  SEMs. n.s. not significant.

Next, we would seek the upstream regulator for SENP1-Sirt3 activation during T cell memory development. Since AMPK signalling promotes memory T cell generation^14,16,23^, we thus speculated that AMPK would trigger the activation of SENP1-Sirt3 axis during memory development. Indeed, we observed that an AMPK agonist metformin treatment increased the mitochondrial SENP1, decreased the SUMOylated Sirt3, and reduced mitochondrial acetylation in T cells (Fig. 2a–d, and Supplementary Fig. 2a), suggesting that AMPK is an upstream regulator to trigger SENP1-Sirt3 signalling in T cells.

We further explored the physiological relevance of AMPK activation of SENP1-Sirt3 axis in T cells. It is known that the clonal expansion of effector T cells accelerates glucose consumption in effector sites, which results in glucose limitation at the late phase of immune response^16,22^. Further investigation showed that glucose limitation activated SENP1-Sirt3 signalling in mitochondria^31,32^. Therefore, we reasoned that glucose limitation would be a physiological signal to trigger AMPK coupled SENP1-Sirt3 signalling during effector-memory T cell differentiation. To testify this, culture medium containing 2 mM glucose was applied to mimic low-glucose condition^42^, and low glucose markedly increased the memory cell population in effector T cell culture condition at day 6 upon antigen stimulation (Fig. 2e and Supplementary Fig. 2b, c). Moreover, low glucose activated AMPK in effector T cells shown as increased phosphorylation of the AMPK substrate acetyl-CoA carboxylase (ACC) (Fig. 2f). Furthermore, low glucose induced mitochondrial SENP1, reduced mitochondrial Sirt3 SUMOylation and decreased mitochondrial protein acetylation in CD8^+^ effector T cells (Fig. 2f, g). Importantly, Compound C, an AMPK inhibitor, reversed these low-glucose-triggered activates in effector T cells (Fig. 2e–g), suggesting that the glucose limitation triggers SENP1-Sirt3 signalling via AMPK to promote T effector-memory differentiation.

### FBP suppresses AMPK coupled SENP1-Sirt3 signaling in memory T cells

AMPK is capable of sensing glucose availability and regulates metabolic adaption and effector responses^42^. In the early stage of immune response, glucose is available at effector site, which maintains low AMPK activation in these early-activated effector cells. The effectors would suffer from limitation of glucose and the other nutrients at effector site in the late phase of immune response, which activates AMPK in these late effectors^16,22^. Interestingly, it has been demonstrated that late effector T cells exhibit much lower glycolysis activity than the early effectors do^22,43^, suggesting that the production of glycolytic metabolites such as fructose-1,6-bisphosphate (FBP) might be decreased in the late phase of immune response. In fact, we showed that FBP production in T cells cultured in low glucose containing medium markedly reduced (Supplementary Fig. 2e). Since FBP has been shown to repress AMPK activation in glucose starvation^42^, we proposed that glucose limitation may trigger AMPK coupled SENP1-Sirt3 signaling via reducing FBP production during effector-memory T cell differentiation. To prove this hypothesis, the effector T cells were cultured in regular or low glucose containing media supplemented with FBP (5 mM) for 3 days (Supplementary Fig. 2d–f)^44,45^. FBP addition markedly reduced the low glucose effects on AMPK activation via an AMP/ADP-independent mechanism (Supplementary Fig. 2f), and inhibited memory development from Sirt3 WT effector T cells (Fig. 2h). Notably, FBP had no effect on memory development from Sirt3 K223R T_E_ cells even under low-glucose condition (Fig. 2h, Supplementary Fig. 2g). We further determined whether FBP affect the AMPK coupled SENP1-Sirt3 signalling in T cells. As shown in Fig. 2i, FBP blocked low-glucose-induced AMPK phosphorylation. FBP treatment also prevented low-glucose-induced mitochondrial SENP1 level and reduction of Sirt3 SUMOylation in cultured CD8^+^ T_E_ cells. Low-glucose-induced mitochondrial protein deacetylation was also reversed in FBP treated CD8^+^ T_E_ cells (Fig. 2i, j).

To further determine the role of SENP1-Sirt3 signaling in AMPK-mediated T memory development, we analysed T memory cell development in *Senp1* cKO T cells. *Senp1* deficiency decreased T memory cell development under normal glucose condition. Low glucose or metformin treatment could not increase the population of *Senp1* cKO T memory cells as compared to *Senp1* WT (Fig. 2k and Supplementary Fig. 2h–j). Moreover, we did not observe a change in SUMOylated Sirt3 as well as mitochondrial acetylation in the mitochondria of *Senp1* cKO T cells responding to low glucose or metformin treatment as compared to *Senp1* WT control T cells (Fig. 2l–n). However, the mitochondrial acetylation in Sirt3 K223R CD8^+^ T cells was lower than that in Sirt3 WT control cells, and was maintained under low glucose or metformin treatment (Fig. 2o–q). These data suggest that glucose limitation may trigger AMPK coupled SENP1-Sirt3 signalling in T cell memory development.

### SENP1-Sirt3 axis enhances the survival of memory T cells

**Fig. 3:**
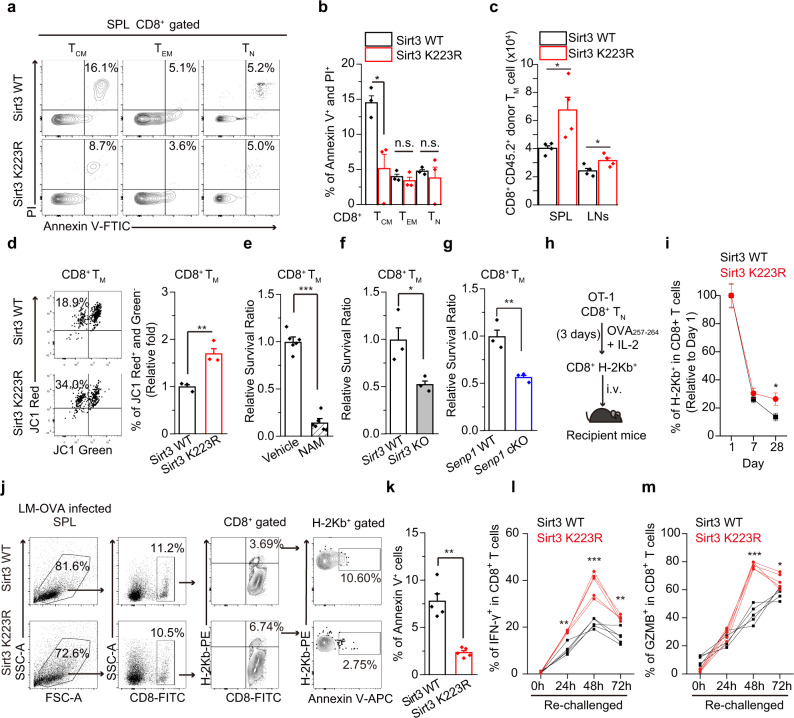
SENP1-Sirt3 axis enhances the survival of T_M_ cells. **a**, **b** The apoptotic ratios of CD8^+^ T_CM_, T_EM_ and T_N_ cells from Sirt3 WT or K223R mouse splenocytes were measured (*n* = 3 mice, *P = 0.028). **c** T_M_ cells (1.5×10^6^, CD45.2) generated from Sirt3 WT or K223R mice were injected into congenic recipient mice (CD45.1). The donor T_M_ cell numbers in the recipient spleen (SPL) or lymph nodes (LNs) were analysed on day 2 (*n* = 4 mice, *P = 0.042 in SPL and *P = 0.046 in LN). **d** T_M_ cells generated from Sirt3 WT or K223R mice were analysed by JC-1 staining. (*n* = 3 biologically independent samples, **P = 0.0083). **e** The survival ratio of T_M_ cells treated with NAM (5 mM) on day 3 was analysed (*n* = 6 biologically independent samples, ***P < 0.0001). **f** The survival ratio of T_M_ cells from *Sirt3* WT or *Sirt3*-KO mice was detected (*n* = 3 biologically independent samples, *P = 0.047). **g** The survival ratio of T_M_ cells from *Senp1* WT or cKO mice was analysed (*n* = 3 biologically independent samples, **P = 0.0089). **h**, **i** The activated OT1 T cells (H-2Kb^+^) were transplanted into congenic recipient mice (1.0 × 10^7^/mouse) (**h**). The H-2Kb^+^ populations in the recipient blood were analyzed (*n* = 7 mice, *P = 0.024) (**i**). **j**, **k** The apoptotic ratio of spleen CD8^+^ H-2Kb^+^ cells from recipients was quantified after 21 days LM-OVA infection in vivo (*n* = 5 mice, **P = 0.0016).). **l**, **m** The populations of IFN-γ (**l**)**-** or Granzyme B (GZMB) (**m**)-positive T cells in T_M_ cells were analysed after re-challenge (*n* = 5 mice, **P = 0.0013, ***P ≤ 0.001, **P = 0.0033 at 24, 48 and 72 h, respectively, for IFN-γ; ***P ≤ 0.0001 and *P = 0.029 at 48 h and 72 h, respectively, for GZMB). Two-tailed unpaired *t*-test (**b**–**g**, **k**). Two-way ANOVA followed by Fisher’s LSD test (**i**, **l**, **m**). All data are shown as the means ± SEMs. n.s. not significant.

We next explored the mechanism underlying SENP1-Sirt3 axis promoting T cell memory development. It is well-known that cell survival is a critical feature gained for T cell memory development^46,47^. There were much less apoptotic central memory T cells (T_CM_, CD8^+^ CD62L^+^ CD44^+^) in the spleens of Sirt3 K223R mutant mice than that of Sirt3 WT mice (Fig. 3a, b), suggesting that SENP1-Sirt3 axis may regulate T_M_ cell survival. In vivo survival assay using T cell transplantation model also showed that the Sirt3 K223R donor T_M_ cells in the spleens (SPL) or lymph nodes (LNs) of the recipient mice were markedly more abundant than Sirt3 WT donor T_M_ cells in the recipient mice (Fig. 3c). Additionally, Sirt3 K223R T_M_ cells exhibited a higher mitochondrial membrane potential (MMP), an indicator of mitochondrion-dependent apoptosis, than Sirt3 WT T_M_ cells did (Fig. 3d), suggesting that SENP1-Sirt3 axis may protect mitochondria against apoptotic challenges. Moreover, treating T_M_ cells with the sirtuin inhibitor nicotinamide (NAM) (Fig. 3e) or the knockout of *Sirt3* in T_M_ cells markedly decreased the in vitro survival of T_M_ cells (Fig. 3f and Supplementary Fig. 3a). Consistently, *Senp1* cKO T_M_ cells also decreased survival compared with *Senp1* WT cells (Fig. 3g).

We generated an OT1 T cell transplantation model to investigate the role of SENP1-Sirt3 axis in T cell survival in vivo. Sirt3 WT or Sirt3 K223R OT1 tetramer^+^ (H-2Kb^+^) T cells were transplanted into congenic recipient mice (Supplementary Fig. 3b, c). The population of Sirt3 K223R CD8^+^ H-2Kb^+^ T cells was slightly larger than that of Sirt3 WT CD8^+^ H-2Kb^+^ T cells at day 7 post-transplantation. However, much more abundance of Sirt3 K223R CD8^+^ H-2Kb^+^ T cells (assumed memory T cells) than Sirt3 WT CD8^+^ H-2Kb^+^ T cells was observed in the recipient mice at day 28 post-transplantation (Fig. 3h, i). Furthermore, Sirt3 K223R CD8^+^ H-2Kb^+^ splenocytes showed much less apoptotic ratio (Annexin V^+^) than Sirt3 WT CD8^+^ H-2Kb^+^ splenocytes did at day 21 after LM-OVA infection (Fig. 3j, k). Additionally, Sirt3 K223R OT1 T_M_ cells displayed more IFN-γ- and Granzyme B-positive T cells than Sirt3 WT-OT1 T_M_ cells upon OVA_257-264_ peptide re-challenge (Fig. 3l, m, and Supplementary Fig. 3d–f). Thus, we propose that SENP1-Sirt3 axis may be a positive signalling pathway that promotes T cell survival leading memory development.

### SENP1-Sirt3 axis promotes OXPHOS and mitochondrial fusion in T_M_ cells

**Fig. 4:**
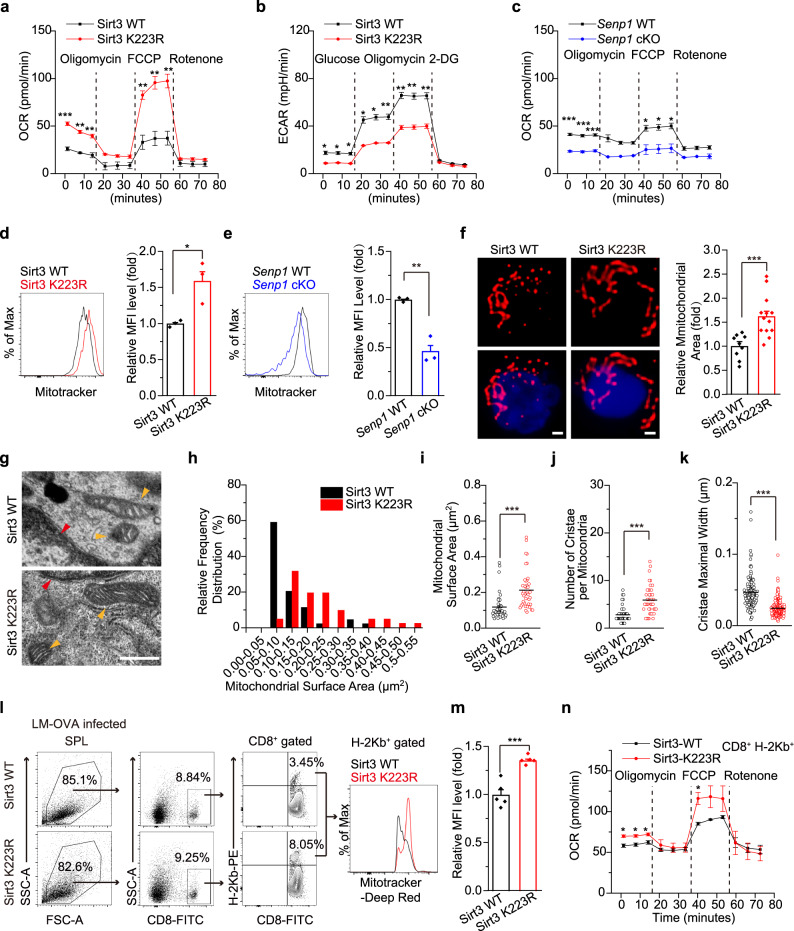
The SENP1-Sirt3 axis promotes OXPHOS and mitochondrial fusion in T_M_ cells. **a**, **b** The OCR (**a**) and ECAR (**b**) of Sirt3 WT or K223R T_M_ cells (*n* = 3 mice, ***P = 0.0006, **P = 0.0022, **P = 0.0046, **P = 0.0085, **P = 0.0044 **P = 0.0042 at 1.3, 7.8, 14.3, 40.5, 47.0 and 53.5 min, respectively, (**a**); *P = 0.015, *P = 0.016, *P = 0.013, *P = 0.015, *P = 0.01, **P = 0.0093, **P = 0.002, **P = 0.002 **P = 0.0021 at 1.3, 7.9, 14.4, 21.1, 27.7, 34.2, 40.9, 47.5 and 54.2 min, respectively (**b**). **c** The OCR of *Senp1* WT or cKO T_M_ cells (*n* = 3 mice, ***P = 0.0005, ***P = 0.0007, ***P = 0.0006, *P = 0.027, *P = 0.02 *P = 0.018 at 1.3, 7.9, 14.6, 41.1, 47.7 and 54.2 min, respectively). **d**, **e** The MitoTracker staining of T_M_ cells from Sirt3 WT or K223R (*n* = 3 mice, *P = 0.025) (**d**), and *Senp1* WT or cKO (*n* = 3 mice, **P = 0.0026) (**e**). **f** The mitochondrial area of Sirt3 WT or K223R T_M_ cells (*n* = 9 cells for WT, *n* = 13 cells for K223R, ***P = 0.0008). The scale bars, 1 μm. **g**–**k** The TEM of mitochondria in Sirt3 WT or K223R T_M_ cells. The red arrows, nucleus. The orange arrows, mitochondria. The scale bars, 0.5 μm (**g**). The mitochondrial surface area (*n* = 44 mitochondria from WT, *n* = 41 mitochondria from K223R, ***P < 0.0001) (**h**–**i**), the cristae number per mitochondria (***P < 0.0001) (**j**), and the maximal cristae width (*n* = 124 cristae from WT, *n* = 242 cristae from K223R, ***P < 0.0001) (**k**) were analyzed. **l**–**n** The MitoTracker staining (*n* = 5 mice, ***P = 0.0004) (**l**, **m**), the OCR (*n* = 3 biologically independent samples, *P = 0.011, *P = 0.016, *P = 0.031, *P = 0.047 at 1.3, 7.7, 14.2 and 40.2 min, respectively) (**n**) of splenic CD8^+^ H-2Kb^+^ cells after LM-OVA infection. Two-tailed unpaired *t*-test (**d**–**f**, **i**–**k**, **m**). Two-way ANOVA followed by Fisher’s LSD test (**a**–**c**, **n**). All data are shown as the means ± SEMs. n.s. not significant.

To understand the metabolic basis of SENP1-Sirt3 axis in promoting the survival of memory T cells, we measured the oxygen consumption rate (OCR) and the extracellular acidification rate (ECAR) in T_M_ cells. Sirt3 K223R T_M_ cells displayed a higher OCR but a lower ECAR than Sirt3 WT T_M_ cells did (Fig. 4a, b). Consistently, OCR in *Senp1*-deficient T_M_ cells was lower than that in *Senp1* WT T_M_ cells (Fig. 4c). In addition, Sirt3 K223R T_M_ cells had fewer long-chain acylcarnitine species and higher levels of ATP and Acetyl-CoA in mitochondria than Sirt3 WT T_M_ cell mitochondria (Supplementary Fig. 4a–c). These data suggest that the SENP1-Sirt3 signalling may promote FAO-fuelled OXPHOS in T_M_ cells.

Mitochondrial fusion is required for the increase in OXPHOS activity linked to T_M_ cell development^7^. Thus, we tested whether the SENP1-Sirt3 axis promotes mitochondrial fusion in T_M_ cells. As shown in Fig. 4d, f, Sirt3 K223R T_M_ cells presented increased mitochondrial mass and larger mitochondrial area as compared to Sirt3 WT T_M_ cells. *Senp1* deficiency reduced the mitochondrial mass in T_M_ cells compared with *Senp1* WT accordingly (Fig. 4e). Furthermore, the mitochondrial size and surface area in Sirt3 K223R T_M_ cells were higher than those in Sirt3 WT cells (Fig. 4g–i). Sirt3 K223R T_M_ cells showed more cristae per mitochondria and a decreased maximal cristae width than Sirt3 WT T_M_ cells (Fig. 4j, k). The mitochondrial mass and OCR were more increase in Sirt3 K223R CD8^+^ H-2Kb^+^ cells from the spleen of recipients than that in Sirt3 WT control at day 21 after LM-OVA infection (Fig. 4l–n). These data indicate that the SENP1-Sirt3 axis may enhance mitochondrial fusion and mitochondrial inner membrane remodelling in T_M_ cells.

### YME1L1 is a substrate of Sirt3 in OPA1 processing and mitochondrial fusion

**Fig. 5:**
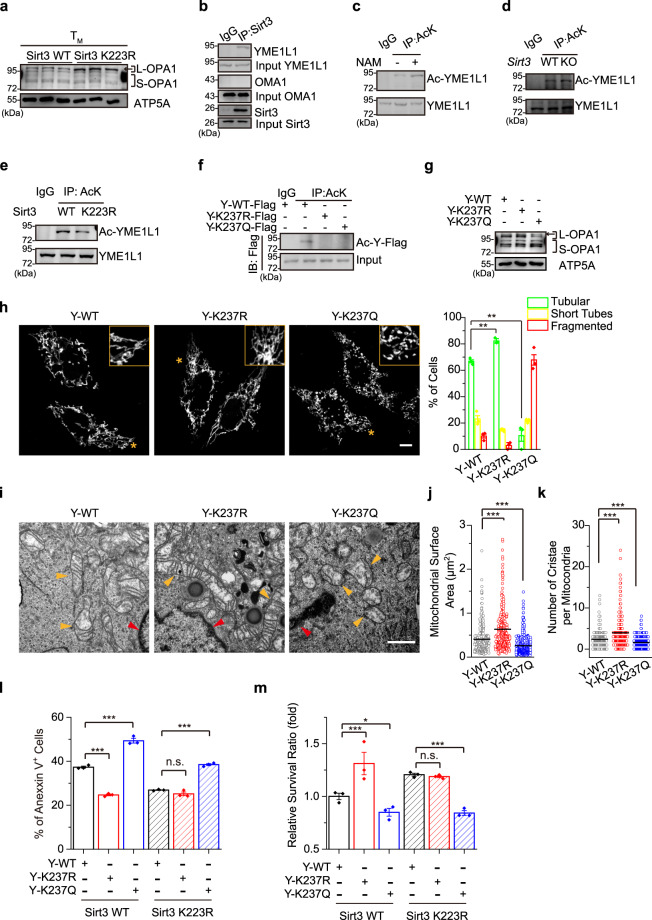
Sirt3 regulates YME1L1-mediated OPA1 processing and mitochondrial fusion. **a** Mitochondrial lysates from Sirt3 WT or K223R T_M_ cells were blotted with anti-OPA1 antibody (*n* = 3 biologically independent samples). **b** Mitochondrial lysates from Jurkat cells were immunoprecipitated with anti-Sirt3 antibody and blotted with anti-YME1L1 and -OMA1 antibodies. **c**–**f** YME1L1 acetylation level in NAM (5 mM, 8 h) treated Jurkat cells (**c**), Sirt3 WT or KO T_M_ cells (**d**), Sirt3 WT or K223R T_M_ cells (**e**), in Flag-YME1L1 WT (Y-WT), YME1L1 K237R (Y-K237R) or YME1L1 K237Q (Y-K237Q) plasmids transfected 293 T cells (**f**). **g** OPA1 protein in HeLa cells infected with Y-WT, Y-K237R or Y-K237Q lentivirus. **h** The percentages of HeLa cells with tubular, short-tube-shaped or fragmented mitochondria were determined with MitoTracker staining (*n* = 3 independent experiment, **P = 0.0012, Y-WT vs Y-K237R; **P = 0.0056, Y-WT vs Y-K237Q for tubular). The area denoted by the asterisk is enlarged on the top right, and the scale bars, 10 μm. **i**–**k** The mitochondrial microscopy of Jurkat cells infected with Y-WT, Y-K237R or Y-K237Q lentivirus (**i**). The mitochondrial surface area (**j**) and the number of cristae per mitochondria (**k**) (Y-WT, *n* = 282 mitochondria; Y-K237R, *n* = 319 mitochondria; Y-K237Q, *n* = 474 mitochondria) were analysed (***P < 0.0001, Y-WT vs Y-K237R; ***P < 0.0001, Y-WT vs Y-K237Q (**j**); ***P < 0.0001, Y-WT vs Y-K237R; ***P < 0.0001, Y-WT vs Y-K237Q (**k**)). The scale bars, 1 μm. **l**, **m** Sirt3 WT or K223R T_M_ cells were infected with lentivirus (Lv) containing Y-WT, Y-K237R or Y-K237Q (*n* = 3 biologically independent samples). The apoptotic ratio (***P < 0.0001, Y-WT vs Y-K237R; ***P < 0.0001, Y-WT vs Y-K237Q in Sirt3 WT; ***P < 0.0001, Y-WT vs Y-K237Q in Sirt3 K223R) (**l**), and the survival ratio (***P = 0.0007, Y-WT vs Y-K237R; *P = 0.0499, Y-WT vs Y-K237Q in Sirt3 WT; ***P = 0.0002, Y-WT vs Y-K237Q in Sirt3 K223R) (**m**) were measured. All immunoblots are representative of three independent experiments (**a**–**g**). Two-way ANOVA followed by Fisher’s LSD test (**h**, **j**–**m**). All data are shown as the means ± SEMs. n.s. not significant.

The mitochondrial inner membrane protein OPA1 is a master regulator of mitochondrial inner membrane fusion and cristae remodelling^48–52^. There are several OPA1 isoforms in mammalian cells. Long-form OPA1 (L-OPA1) is located in the inner membrane and promotes mitochondrial inner membrane fusion^21,49^. L-OPA1 is cleaved at two distinct sites to short-form OPA1 (S-OPA1), which is released into the intermembrane space, by two proteins called yeast mitochondrial AAA metalloprotease YME1L1 and metalloendopeptidase OMA1^19,48,49^. Although S-OPA1 is dispensable for fusion, its accumulation promotes mitochondrial fission^48^. To determine whether the SENP1-Sirt3 axis modulates OPA1-mediated mitochondrial fusion in T_M_ cells, levels of OPA1 isoforms in Sirt3 K223R and WT cells were measured. We found that Sirt3 K223R T_M_ cells had higher levels of mitochondrial L-OPA1 than Sirt3 WT cells (Fig. 5a), which indicated that the SENP1-Sirt3 axis suppresses OPA1 cleavage.

Sirt3 can interact with YME1L1 but not OMA1^53^ (Fig. 5b). We further identified YME1L1 as an acetylated protein (Supplementary Fig. 5a) and thus hypothesized that Sirt3 might deacetylate YME1L1 to modulate OPA1 cleavage. NAM-treated Jurkat T cells or Sirt3-knockout T_M_ cells increased YME1L1 acetylation (Fig. 5c, d). Sirt3 K223R T_M_ cells reduced YME1L1 acetylation as compared to Sirt3 WT cells (Fig. 5e). Furthermore, we mapped the K237 site as a unique acetylation residue on YME1L1 (Fig. 5f and Supplementary Fig. 5a). The K237R mutant impaired the ability of YME1L1 to process OPA1 cleavage (Fig. 5g) and subsequently promoted mitochondrial fusion (Fig. 5h–k). In contrast, YME1L1 K237Q mutant mimicking its acetylated form promoted OPA1 processing, resulted in a decrease in mitochondrial fusion compared with YME1L1 WT and K237R (Fig. 5h–k, and Supplementary Fig. 5b). These data reveal that Sirt3 deacetylates YME1L1 leading to suppression of OPA1 processing, which promotes mitochondrial fusion.

To determine whether YME1L1 acetylation affects T_M_ cell survival, we overexpressed YME1L1 mutants in either Sirt3 WT or K223R T_M_ cells by lentiviral transduction. As shown in Fig. 5l, m, YME1L1 K237R expression significantly reduced the apoptotic cell numbers in Sirt3 WT T_M_ cells but not in Sirt3 K223R T_M_ cells. In contrast, YME1L1 K237Q induced the apoptosis of both Sirt3 WT and K223R T_M_ cells (Fig. 5l, m, and Supplementary Fig. 5c). These data indicate that the Sirt3-mediated deacetylation of YME1L1 promotes T_M_ cell survival and T cell memory development.

### Sirt3 K223R enhances the anti-tumour immunity of CD8^+^ T cells

**Fig. 6:**
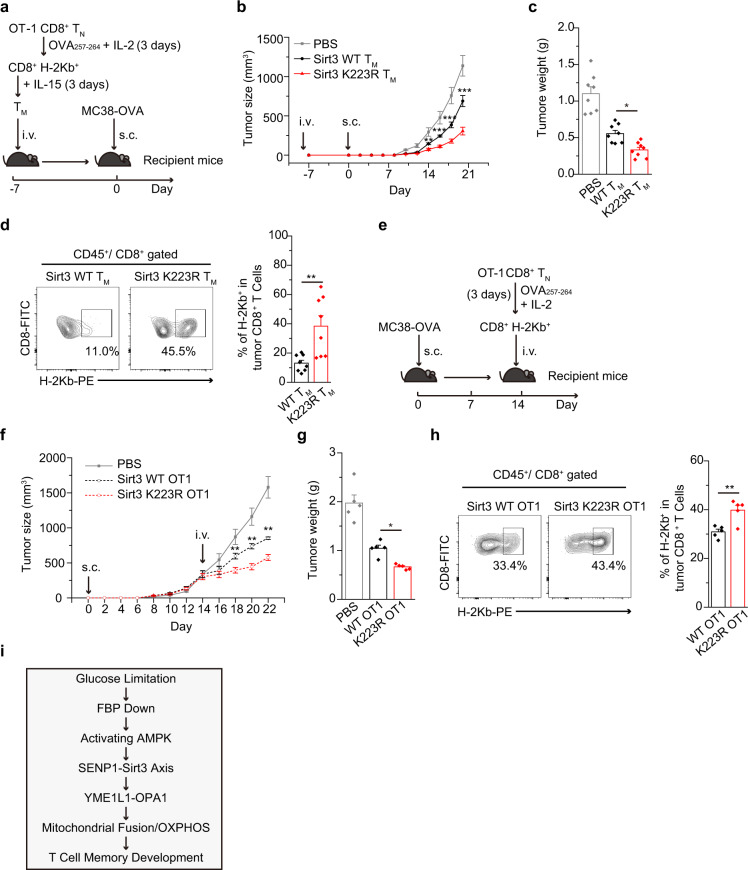
Sirt3 K223R enhances the anti-tumour immunity of CD8^+^ T cells. **a–d** Sirt3 WT or K223R OT1 T_M_ cells (1.5 × 10^6^) were transplanted into congenic recipient mice (C57BL/6). Seven days’ post-transplantation, MC38-OVA tumour cells (1.0 × 10^5^) were subcutaneously inoculated into the recipient mice (*n* = 8 mice per group) (**a**). The tumour growth curve is shown in (Two-way ANOVA followed by Fisher’s LSD test: **P = 0.0026, ***P < 0.0001, ***P = 0.0005 and ***P = 0.0007 on day 14, day 16, day 18 and day 20, respectively) (**b**). The tumour weight was measured at day 20 post-injection (One-way ANOVA followed by Fisher’s LSD test: *P = 0.021 for Sirt3 WT T_M_ vs Sirt3 K223R WT T_M_) (**c**). The frequency of donor CD8^+^ T cells (H-2Kb^+^) in the tumours of the recipient mice was assessed by FACS (Two-tailed unpaired *t*-test: **P = 0.0041) (**d**). **e**–**h** MC38-OVA tumour cells (1.0×10^5^) were planted subcutaneously into recipient mice (C57BL/6). OVA_254-264_ activated OT1-T cells (1 × 10^7^) were freshly obtained from Sirt3 WT or K223R OT1 naïve T cells and intravenously (i.v.) transplanted into the above-mentioned mice at day 14 after the injection of tumour cells (*n* = 5 mice per group) (**e**). The tumour growth curve was recorded (Two-way ANOVA followed by Fisher’s LSD test: **P = 0.008, **P = 0.0011 and **P = 0.0013 on day 18, day 20 and day 22, respectively) (**f**). The tumour volume (mm^3^) was calculated using the following equation: (length × width^2^)/2. The tumour weight was measured at day 22 after the injection of tumour cells (One-way ANOVA followed by Fisher’s LSD test: *P = 0.025 for Sirt3 WT-OT1 vs Sirt3 K223R OT1) (**g**). The frequency of donor CD8^+^ T cells (H-2Kb^+^) isolated from the tumours in the recipient mice was determined by FACS analysis (Two-tailed unpaired *t*-test: **P = 0.0047) (**h**). **i** Model depicting the role of the AMPK-SENP1-Sirt3 axis in regulating FAO-fuelled OXPHOS and mitochondrial fusion in CD8^+^ T_M_ cells.

To explore the biological significance of SENP1-Sirt3 signalling in T_M_ cells, we transplanted Sirt3 WT or K223R T_M_ (OT1/H-2Kb^+^) cells into congenic recipient mice. At day 7 post-transplantation, we inoculated MC38-OVA tumour cells into the recipient mice subcutaneously (Fig. 6a) and observed drastically delayed tumour growth in the Sirt3 K223R T_M_ cell-transplanted mice compared with that in the Sirt3 WT T_M_ cell-transplanted mice (Fig. 6b, c). The analysis of tumour-infiltrating CD8^+^ T cells showed that the frequency of Sirt3 K223R donor CD8^+^ T cells (H-2Kb^+^) was higher than that of Sirt3 WT donor CD8^+^ T cells (Fig. 6d). Furthermore, we used OVA-activated OT1 T cells for immune therapy in the established MC38-OVA tumour model (Fig. 6e). As shown in Fig. 6f, g, Sirt3 K223R OT1 T cells were more potent in inhibiting tumour growth than Sirt3 WT-OT1 T cells (Fig. 6f, g). Consistently, Sirt3 K223R T cells (H-2Kb^+^) were markedly more abundant in MC38-OVA tumour tissue than Sirt3 WT T cells (Fig. 6h). These data suggest that activation of the SENP1-Sirt3 axis may potentially promote the anti-tumour immunity of T cells.

## Discussion

In this study, we found that the AMPK coupling SENP1-Sirt3 signalling promotes T cell survival and memory development. Glucose limitation is a physiological signal to stimulate AMPK signalling and further activate SENP1-Sirt3 axis in T cell memory development. A mechanistic analysis revealed that SENP1-Sirt3 signalling promoted both OXPHOS activity and mitochondrial fusion through reducing YME1L1-mediated OPA1 cleavage in T_M_ cells. Furthermore, we showed that glycolytic metabolite FBP suppressed AMPK coupled SENP1-Sirt3 signalling in T cells. Therefore, these data reveal that glucose limitation promotes AMPK-SENP1-Sirt3 signalling and T cell memory development via reducing FBP production (Fig. 6i).

Limitation of oxygen and nutrients can weaken or even damage the effector function in T cells. This study showed that glucose limitation would be beneficial for T cell memory development. Ma et al. analysed T cell glucose utilization in vivo and found that glucose metabolism in T cells changes dynamically over an immune response^43^. Early effectors undergo clonal expansion and demand glucose for bioenergy and biosynthesis^43^, which causes glucose limitation at effector sites. Late effector T cells show lower biosynthetic glucose metabolism than early effectors do because of lower proliferation rates or glucose limitation^43^. Production of the glycolytic metabolite FBP may change along with the course of immune response due to the glucose metabolic alterations. Early effector T cells possess increased glycolysis and FBP production, which suppresses AMPK coupled SENP1-Sirt3 signalling at this status. At the late phase, glycolysis is markedly reduced in effector T cells, resulted in downregulated FBP production, which releases the inhibition from AMPK coupled SENP1-Sirt3 signalling for memory development. We thus reveal a critical role of FBP as a metabolite in controlling T cell fate during immune response.

AMPK is a crucial regulator in effector T cell metabolic adaptation to nutrient starvation^22^. AMPK signalling in effector T cells downregulates mTORC1 activity, which in turn diminishes energy expenditure and activates glutamine-dependent OXPHOS instead of glucose for ATP production^22^. Compared with that in effector cells, AMPK activation is increased in memory T cells^14,23,54^. We demonstrated that glucose limitation induced AMPK activation, which activated SENP1-Sirt3 signalling for T cell memory development at late phase of effector immune response. SENP1-Sirt3 signalling rescued AMPK inhibition-diminished memory development, suggesting that SENP1-Sirt3 signalling is a crucial downstream event following AMPK activation in memory development.

We revealed that the SENP1-Sirt3 axis activated OXPHOS and mitochondrial fusion in T cells, and this activation increased T cell survival and memory development. Buck et al. reported that OPA1-mediated mitochondrial fusion was required for T_M_ cell development^7^. YME1L1 and OMA1 are two proteases that cleave L-OPA1 to generate S-OPA1^48,49^. In this study, we identified YME1L1 but not OMA1 as a critical substrate of Sirt3 for deacetylation to promote mitochondrial fusion. YME1L1 acetylation mutant represented inhibited SENP1-Sirt3 axis in modulating mitochondrial fusion and memory T cell survival, suggesting that deacetylation of YME1L1 by SENP1-Sirt3 signalling is a critical regulation mechanism in controlling OPA1 cleavage and subsequent OPA1-mediated mitochondrial fusion in T cell memory development. Meanwhile, the increased cristae in SENP1-Sirt3-mediated mitochondrial fusion provide a structural basis for high OXPHOS activity shown in memory T cells. Therefore, YME1L1 may play a crucial role in OPA1-mediated mitochondrial fusion and T cell memory development.

Our findings may contribute to rescuing T cell dysfunction in the tumour microenvironment. In solid tumours, tumour cells consume many more nutrients to meet their proliferation demands, which results in the depletion of glucose and other nutrients in the tumour microenvironment^17,18^. This unique feature of the tumour microenvironment can suppress T cell activity^17^ and also limits its anti-tumour immune efficacy^18^. In this study, we found that SENP1-Sirt3 signalling promoted T cell infiltration into tumour tissue and enhanced anti-tumour immunity. We also showed that metformin activated AMPK-SENP1-Sirt3 signalling in T cells. Therefore, we hypothesize that the activation of AMPK-SENP1-Sirt3 signalling in T cells is a potent strategy for anti-tumour therapy. It is expected that this strategy combined with anti-PD-1/PD-L1 immunotherapy may induce the anti-tumour immunity of T cells.

## Methods

### Mice

C57BL/6 Sirt3 wild-type and K223R mice were described in our previous studies^31^. C57BL/6 CD45.1 mice (Jackson Laboratory), *Cd4*-*Cre* mice (Jackson Laboratory) and major histocompatibility complex (MHC) class I-restricted OVA-specific TCR OT1 transgenic mice (Jackson Laboratory) were obtained from Shanghai Institute of Immunology^11^. C57BL/6 Sirt3-knockout (Sirt3 KO) mice were obtained from Prof. Shimin Zhao’s Laboratory (Fudan University, China)^55^. Sirt3 WT or K223R mice were crossed to C57BL/6 OT1 transgenic mice to generate Sirt3 WT or K223R OT1 mice. To create T cell-specific *Senp1* cKO mice (*Senp1* ^*flox/flox*^ × *Cd4-Cre*), *Senp1* ^*flox/flox*^ mice were crossed with *Cd4-Cre* mice. *Senp1* ^*flox/flox*^ mice produced in previous studies^56,57^. All mice were bred and maintained under specific pathogen free (SPF) conditions. All mice were bred and maintained under a 12 h reverse light/dark cycle with a temperature of 22 ± 2 °C and a humidity of 40–70%. Age and sex matched male and female adult (6–8 week old) mice were used in each independent experiment. The animal experiments were performed in strict accordance with the “Guide for the Care and Use of Laboratory Animals”, which were approved by the Experimental Animal Ethical Committee at Shanghai Jiao Tong University School of Medicine.

### Cell culture

HEK-293T (ATCC CRL-11268), HeLa (ATCC CCL-2) and OVA-expressing mouse MC38 (MC38-OVA) cells were cultured in Dulbecco’s modified Eagle’s medium (DMEM, 4500 mg/l glucose, 4 mM L-glutamine). Jurkat Clone E6-1(ATCC TIB-152) cells were cultured in RPMI 1640, containing 10% fetal bovine serum (FBS), 100 unit/ml penicillin and 100 μg/ml streptomycin. MC38-OVA cells were made by Yang-Xin Fu Lab (UT South western Medical Center, USA)^58^, and were obtained from Shanghai Institute of Immunology. All of the cell lines are mycoplasma free. *YME1L1*-knocked out HeLa cells were generated by CRISPR-Cas9 technology with a specific gRNA (ggaaccgaccatattacaacagg).

### Plasmids

We used primers 5’-atatgcggccgcatgttttccttgtcgagcac-3’ (sense) and 5’-atatgcggccgctatatctcacttccaac-3’ (anti-sense) to amplify *YME1L1* from Human cDNA, and constructed YME1L1-Flag in pcDNA3.1 (+) and pCDH-GFP using standard PCR-based cloning strategies. We mutated YME1L1 K237 to R with primers 5’-ccatcattcgtgagggggtttcttttg-3’ (sense) and 5’-caaacgaaaccccctcacgaatgatgg-3’ (anti-sense), and mutated K237 to Q with primers 5’-ccatcattcgtgcaggggtttcttttg-3’ (sense) and 5’-caaacgaaacccctgcacgaatgatgg-3’ (anti-sense). All plasmids were verified by DNA sequencing. We used pCDH lentivirus system (System Biosciences, CD511B-1) to produce lentiviral-GFP-YME1L1, -YME1L1 K237R, or -YME1L1 K237Q virus according to the manufacture manual.

### T cell culture and activation

CD8 ^+^ OT1 naive T cells were isolated from splenocytes. For T_E_ or T_M_ cell development, these naive T cells were activated with OVA_257–264_ peptide (SIINFEKL, Sigma) or anti-CD3 (5 μg/ml)/anti-CD28 (2 μg/ml), with IL-2 (100 U/ml, PeproTech) for 3 days and then cultured in the presence of either IL-2 (100 U/ml, for T_E_) or IL-15 (10 ng/ml, for T_M_) for another 3 days in T cell media (RPMI 1640 + 10% FBS + 100 U/ml penicillin/streptomycin + 2  mM L-glutamine + 55 μM β-mercaptoethanol)^7,13–15^. For in vitro T_M_ cell survival assays^7,13–15^, T_M_ cell was cultured in 96-well (1 × 10^5^ cells/well) round bottom plates in T cell medium without IL-15 for 3 days, respectively^15^. Using 7-AAD staining analyzed the survival ratio^15^. For differentiation of mouse Th1, Th2, Th17 and Treg cells from CD4 ^+^ naïve T cells in vitro (*n* = 3 biologically independent samples), purified CD4 ^+^ naïve T cells (by EasySep Mouse Naive CD4 ^+^ T Cell Isolation Kit, STEMCELL, Cat#19765) were stimulated by anti-CD3/CD28 + IL-2 and cultured in the presence of polarizing cytokines for 6 days: IL-12 (20 ng/mL, PeproTech, Cat#210-12) + anti-IL-4 (10 ng/mL, Biolegend, Cat#504101), for Th1 cells; or IL-4 (100 ng/mL, PeproTech, Cat#214-14) + anti-IFN-γ (10 ng/mL, Biolegend, Cat#517903) and anti-IL-12(10 ng/mL, Biolegend, Cat#505303), for Th2 cells; or TGF-β1 (5 ng/mL, R&D System, Cat#7666-MB-005) + anti-IL-4 & anti-IFN-γ (10 ng/mL), for Treg cells; or TGF-β1(1 ng/mL) + IL-6 (100 ng/mL, PeproTech, Cat#216-16) + anti-IL-4 & anti-IFN-γ (10 ng/mL), for Th17 cells.

### T cell adoptive transfer

For in vivo T_M_ cell survival assays^7,13–15^, OT1 CD8^+^ T_M_ cells from donor splenocytes (CD45.2) were isolated and transferred intravenously (i.v.) into congenic recipient mice (C57BL/6, CD45.1). For anti-tumor immunity assays, the CD8^+^ T_M_ cells or OVA_257-264_ activated OT1 T cells (H-2Kb^+^, C57BL/6) were transferred intravenously (i.v.) into congenic recipient mice (C57BL/6)^7^.

### LM-OVA infection

*Listeria monocytogenes* expressing-OVA (LM-OVA) was obtained from Dr. Shen’s laboratory^41^, and cultured in Brain-Heart Infusion(BHI) medium with 50 μg/ml Streptomycin. CD8^+^ naïve T cells (1.0 × 10^4^) from Sirt3 WT-OT1 and Sirt3 K223R OT1 were adoptive transferred into congenic recipient mice (C57BL/6). The recipient mice then were either injected (i.v.) with a sub-lethal dose of LM-OVA(1.0 × 10^5^ CFU)^14^.

### T cell lentiviral transduction

Briefly, CD8^+^ T_M_ cells generated from naive T cells using in vitro standard procedure were infected with the packaged lentivirus in the presence of 10 mg/ml polybrene by spinning at 1200 r.p.m. for 90 min at 30 °C^59^. Supernatant was removed after infection and replaced with T cell medium containing IL-15 (10 ng/ml) for 48 h (infection efficient > 80%)^59^. Then we collected the infected T_M_ cells for apoptosis staining and 7-AAD based in vitro survival experiments.

### Mitochondria isolation

The procedure used for mitochondria isolation was described in our previous study^31^. Briefly, cells were incubated in ice-cold isolation buffer TD [135 mM NaCl, 5 mM KCl, 25 mM Tris-HCl (pH 7.5)] containing complete EDTA-free protease inhibitor mixture (Roche). After centrifuging at 600 × *g* for 10 min, the pellet was washed by 10 ml ice-cold TD buffer and centrifuged again at 600 × *g* for 10 min. The pellet was then re-suspended in ice-cold isolation buffer MS containing 210 mM mannitol, 70 mM sucrose, 5 mM Tris-HCl (pH 7.5), 1 mM EGTA, 0.5 mg/ml BSA and protease inhibitor mixture. The lysate was further homogenized in this buffer with a Glass-Teflon motorized homogenizer for 40 times. The mitochondrial fraction was isolated by differential centrifugation 1300 × *g* for 5 min and 17,000 × *g* for 15 min. Subsequently, the pellet was re-suspended in 1.5 M sucrose solution containing 1 mM EGTA and 10 mM Tris-HCl (pH 7.5). A thin layer was formed after gently adding 1 M sucrose solution on the suspension solution. This layer was drawn out from the tube and diluted by 4 volume dilution buffers [1 mM EGTA and 5 mM Tris-HCl (pH 7.5)] to the final concentration of 250 mM sucrose. After centrifugation at 17,000 × *g* for 15 min, the purified mitochondria were dissolved in MS buffer for −80 °C stock.

### Metabolomics

The procedure used for mitochondria metabolomics analysis is described elsewhere^31^. Mitochondrial lysates were precipitated with acetonitrile containing internal standards; the supernatant was separated by centrifugation, and then analyzed by tandem mass spectrometry (TSQ Vantage, Thermo Fisher Scientific). Chromatographic separation was performed on a XBridgeTM Amide column (2.1 × 150 mm, 5 μm; Waters) with a XBridgeTM Amide guard column (2.1 × 10 mm, 5 μm; Waters). The mobile phase was composed of 10 mM ammonium acetate in water (phase A) and acetonitrile (phase B). L-carnitine, acylcarnitines and their internal standards (Sigma–Aldrich) were analyzed in positive ion multiple reaction monitoring (MRM) mode. The raw data files were processed using LCquan 2.7 software (Thermofisher Scientific) to generate chromatographic peak areas of each analyst and their response ratios to internal standards. Then the concentration of C0 and acylcarnitines were calculated from the response ratios and the concentration of their internal standards using isotope dilution method.

### Detection of fructose-1,6-bisphosphate, ATP and acetyl-CoA

Concentration of ATP in T cell mitochondria was determined using an ATP Colorimetric/Fluorometric Assay Kit (Cat#K354-100, Biovision). Concentration of Acetyl-CoA in T cell mitochondria was determined using a PicoProbe^TM^ Acetyl-CoA Fluorometric assay kit (Cat#K317-100, BioVision). Concentration of Fructose-1,6-Bisphosphate (FBP) in pretreated T cells was determined using a PicoProbe Fructose-1,6-Bisphosphate Assay Kit (Cat#K2036, Biovision).

### Flow cytometry

Fluorochrome-labelled antibodies (Biolegend, eBioscience and BD Pharmingen), MitoTracker (Green/Red/Deep Red), or JC-1 (Invitrogen) staining were performed according to the manufacturer’s instructions. OVA-specific CD8^+^ T cells from blood or MC38-OVA tumor were staining with H2-Kb OVA_257-264_ MHC-peptide tetramers (MBL, Japan). Cells were collected on BD FACSVerse^TM^ with BD FACSuite (1.0.5.3841) software or LSR Fortessa X-20 cell analyzer (BD Biosciences) and BD FACSDiva (6.1.3) software, and analyzed using FlowJo (TreeStar) software. CD8^+^ naive T Cells (CD8^+^CD44^-^CD62L^+^) were sorted using a MoFlo Astrios (BeckMan) or isolated by EasySep Mouse Naive CD8^+^ T Cell Isolation Kit (STEMCELL).

### Imaging

For mitochondria observation by confocal fluorescence microscopy, T_M_ cells or HeLa cells were stained with MitoTracker Red (Invitrogen) at 1:2000 dilution for 30 min, followed by the fixation before nuclear counterstaining with DAPI or Hoechst. Images were acquired using a Leica TCS Sp8 STED confocal microscope, and a Leica LAS X Core (3.7.2.22383) software. For transmission electron microscopy, T_M_ or Jurkat cells were fixed in 2.5% glutaraldehyde. Cut sections were imaged using a HITACHI H-7650 or PHILIPS CM-120 transmission electron microscope^31^. RADIUS 2.0 software (EMSIS GmbH, Muenster, Germany) software was used for transmission electron microscope data collection. Image-J (1.52a), Image Pro-Plus (version 6.0), Adobe Photoshop CS6 and Adobe Illustrator CS6 were used to analyse and process fluorescence and electron microscopy images.

### Seahorse analysis

Oxygen consumption rates (OCR) and extracellular acidification rates (ECAR) were measured using a 96-well XF Extracellular Flux Analyzer (EFA) (Seahorse Bioscience) as described as previous study, and Seahorse Wave (2.6.1.53) software was used for data collection^7,15,31^. XF media contains non-buffered RPMI 1640, 25 mM glucose (for OCR), 2 mM L-glutamine and 1 mM sodium pyruvate. One micromolar oligomycin, 1.5 μM fluoro-carbonyl cyanide phenylhydrazone (FCCP), or 100 nM rotenone + 1 μM antimycin A were used in these assays according to the manufacturer’s instructions.

### Statistics and reproducibility

All experiments were performed with at least three biological replicates as indicated in the figure legends. All co-immunoprecipitation and immunoblots were repeated at least three times independently with similar results. Comparisons for two groups were calculated using paired or unpaired two-tailed student’s *t*-tests, as appropriate. And comparisons for more than two groups were calculated using one-way or two-way ANOVA followed by Fisher’s LSD multiple comparison tests, as appropriate. Statistical analysis was performed using GraphPad Prism. The Details of statistical testing and the exact *P-*value are provided in figure legends. The level of significance is indicated as follows: *P < 0.05, **P < 0.01, ***P ≤ 0.001.

### Reporting summary

Further information on research design is available in the Nature Research Reporting Summary linked to this article.

## Supplementary information

Supplementary Information

Reporting Summary

## Data Availability

All data are provided in the article and Supplementary files or are available from the corresponding author on reasonable request. Source data are provided with this paper.

## Source data

Source Data
