# Supplementary material for: Glucose limitation activates AMPK coupled SENP1-Sirt3 signalling in mitochondria for T cell memory development
Source: Nat Commun. 2021 Jul 16;12:4371. doi: 10.1038/s41467-021-24619-2 (PMC8285428; doi:10.1038/s41467-021-24619-2)
Supplement: Supplementary file 1 — Supplementary Information [file 41467_2021_24619_MOESM1_ESM.pdf]

Supplementary Information

Glucose limitation activates AMPK coupled SENP1-Sirt3 signalling in mitochondria for T cell memory development

Jianli He[1,10], Xun Shangguan[2,3,10], Wei Zhou[1,2], Ying Cao[1], Quan Zheng[1], Jun Tu[1], Gaolei Hu[1], Zi Liang[1], Cen Jiang[1], Liufu Deng[3], Shengdian Wang[5], Wen Yang[1], Yong Zuo[1], Jiao Ma[1], Rong Cai[1], Yalan Chen[1], Qiuju Fan[1], Baijun Dong[2], Wei Xue[2], Hongsheng Tan[6], Yitao Qi[7], Jianmin Gu[8], Bing Su[4], Y Eugene Chin[9], Guoqiang Chen[1], Qi Wang[2]*, Tianshi Wang[1]*, Jinke Cheng[1]*

[1] State Key Laboratory of Oncogenes and Related Genes, Renji Hospital Affiliated; Shanghai Key Laboratory for Tumor Microenvironment and Inflammation, Department of Biochemistry and Molecular Cell Biology, Shanghai Jiao Tong University School of Medicine, Shanghai 200025, China.

[2] Department of Urology, Renji Hospital Affiliated, Shanghai Jiao Tong University School of Medicine, Shanghai 200127. China.

[3]Department of Urology, Xinhua Hospital Affiliated to Shanghai Jiao Tong University School of Medicine, Shanghai, 200092, China.

[4] Shanghai Institute of Immunology, Shanghai Jiao Tong University School of Medicine, Shanghai, 200025, China.

[5]Institute of Biophysics, Chinese Academy of Sciences, Beijing, 100101, China

[6]Clinical Research Center, Shanghai Jiao Tong University School of Medicine, Shanghai, 200025, China.

[7]College of Life Sciences, Shaanxi Normal University, Xi'an, Shaanxi, 710119, China.

[8]Department of Thoracic Surgery, Zhongshan Hospital, Fudan University, Shanghai, 200032, China.

[9]Institutes of Biology and Medical Sciences, Soochow University Medical College, Suzhou, Jiangsu, 215123, China.

[10]These authors contributed equally.

* Correspondence and requests for materials should be addressed to Q.W. (email: wqi@sjtu.edu.cn), to T.W. (email: tianshi777@shsmu.edu.cn) or to J.C. (email: jkcheng@shsmu.edu.cn).

Supplementary Figs.

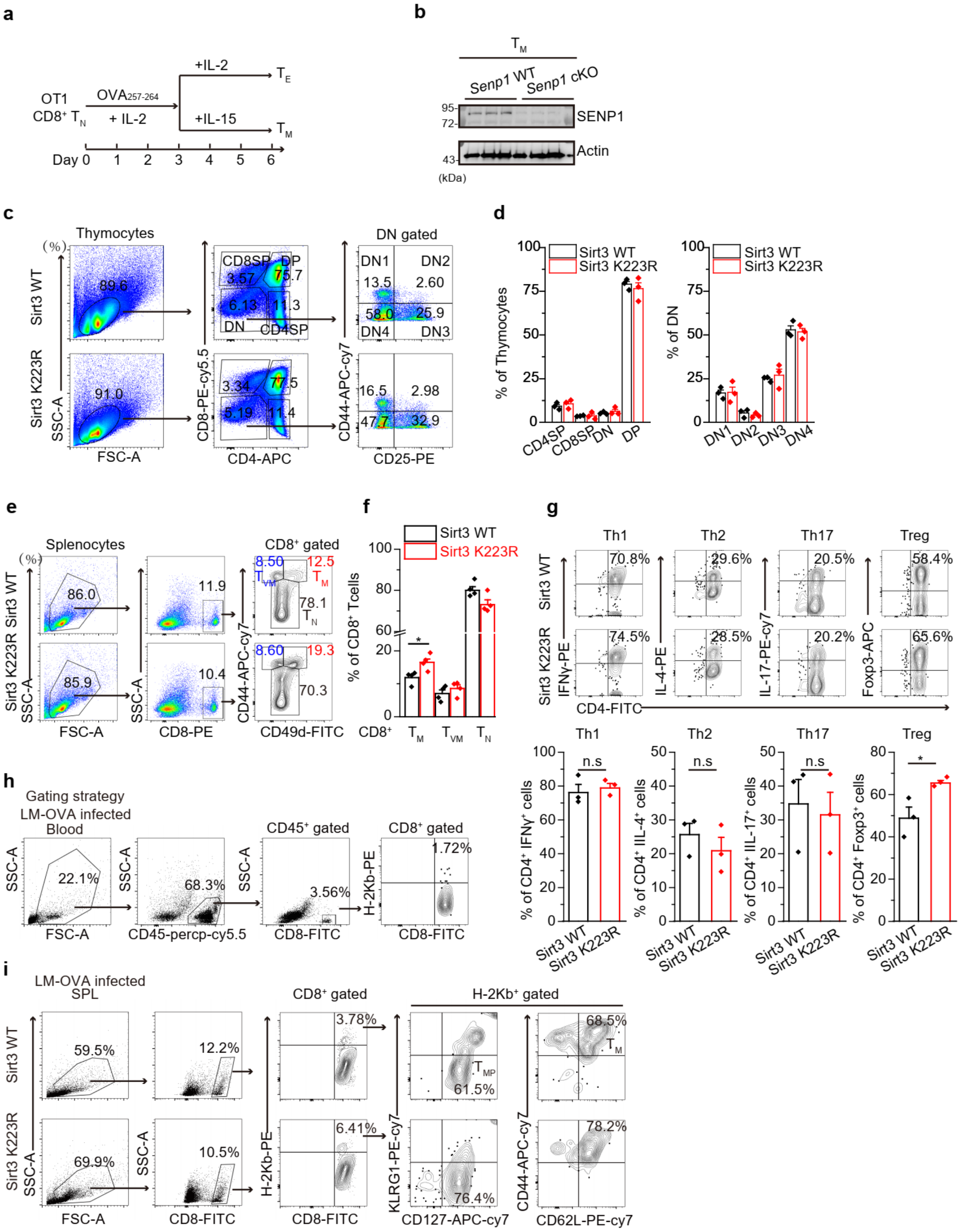

Supplementary Fig. 1 SENP1-Sirt3 axis is activated to promote T cell memory development. a. The generation of effector and memory T cells from naive T cells using *in vitro* standard procedure [1,2]. $CD8^+$ naive OT1 T cells were activated with $OVA_{257-264}$ peptide or anti-CD3/ant-CD28 in the presence of IL-2 for 3 days and subsequently induced in either IL-2 or IL-15 to generate IL-2 induced effector T cells (T_E) and IL-15 induced memory T cells (T_M), respectively; **b.** SENP1 protein in *Senp1*

WT and cKO CD8$^+$ T_M cells (n = 3 mice per group); **c-d.** The analysis of thymocytes from Sirt3 WT and Sirt3 K223R mice was performed to determine the percentage of CD4 and CD8 single-positive (SP) cells, CD4-CD8 double-positive (DP) cells, and CD4-CD8 double-negative (DN) cells within the total thymocyte population and the percentage of different stages of DN (DN1-4) cells. (n = 3 mice per group, Two-tailed unpaired t-test: all $P > 0.05$ in Sirt3 WT vs Sirt3 K223R); **e-f.** A FACS analysis of splenocytes from Sirt3 WT and Sirt3 K223R mice was performed to determine the population of 'true' memory (T_M, CD8$^+$ CD44$^+$ CD49d$^+$), virtual memory (T_{VM}, CD8$^+$ CD44$^+$ CD49d$^-$) and naïve T cells (T_N, CD8$^+$ CD44$^-$ CD49d$^-$) in the CD8$^+$ T cell population (n = 4 mice per group, Two-tailed unpaired t-test: ***P = 0.022 for T_M). Data are shown as mean ± SEM; **g.** The differentiation of Sirt3 WT and K223R CD4$^+$ Th and Treg cells in vitro. Two-tailed unpaired t-test: *P = 0.037 for Treg cells, n.s.: not significant; **h-i.** CD8$^+$ T_N cells from Sirt3 WT-OT1 and Sirt3 K223R-OT1 mice were adoptive transferred into congenic recipient mice, and then the recipients were either injected (i.v.) with LM-OVA**.** The FACS gating strategy for the detection of the antigen-specific CD8$^+$ T cells (OT1 tetramer$^+$, H-2Kb$^+$) in the blood of recipients **(h)**. The FACS gating strategy for the detection of KLRG1$^-$CD127$^+$ memory precursor cells (T_{MP}) and CD44$^+$CD62L$^+$ memory cells (T_M) in the spleen CD8$^+$ H-2Kb$^+$ cells of recipients on day21 post-infection (**i**). The summarizing date were shown in **Fig. 1m, 1n** and **1o** in manuscript.

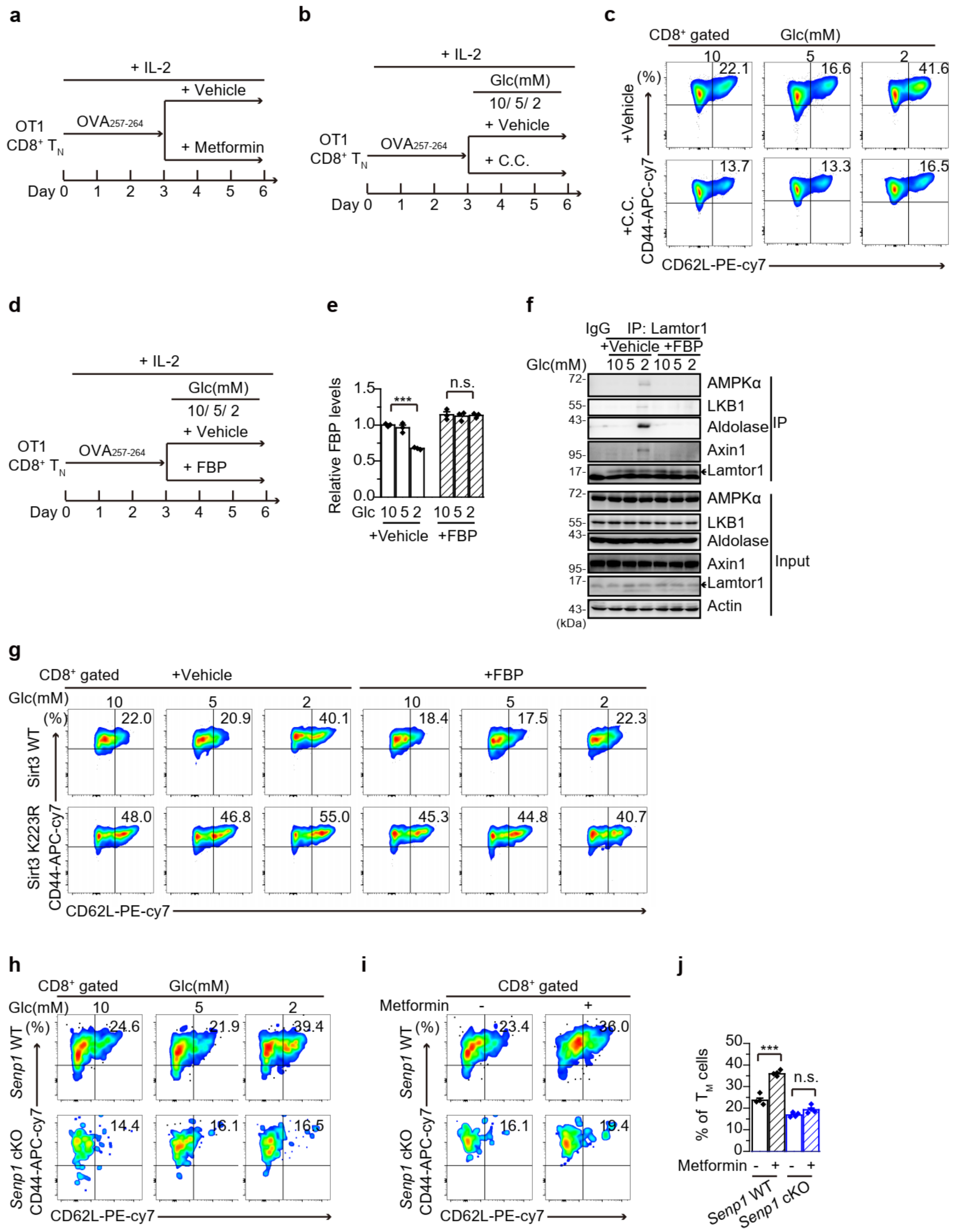

Supplementary Fig. 2 Glucose limitation activates SENP1-Sirt3 axis via AMPK in T_M cells. a. OT1 T_N cells were activated then cultured with metformin (2 mM) for 3 days; **b-c.** OT1 T_N cells were activated and then cultured with gradient concentration of glucose (Glc, 10, 5 and 2 mM) and Compound C (C.C, 10 µM) for 3 days **(b)**. The populations of T_M cells were analyzed (n = 4 biologically independent samples) **(c)**; **d-f.** OT1 T_N cells were activated and then cultured with gradient concentration of glucose and Fructose 1,6-bisphosphate (FBP, 5 mM) treatment for 3 days **(d)**. The relative concentration of FBP was analyzed (n = 3 biologically

independent samples, Two-way ANOVA followed by Fisher's LSD test: ***$P <$ 0.0001 10 mM vs 2 mM Glc culture in vehicle treatment, and 10 mM vs 2 mM Glc culture $P > 0.05$ in FBP treatment) **(e)**. The complex of AMPK/ LKB1/ Aldolase/ Axin1/ Lamtor1 was detected by Co-Immunoprecipitation **(f)**; **g.** OT1 T_N cells were activated and then cultured with gradient concentration of glucose and Fructose 1,6-bisphosphate (FBP, 5 mM) treatment for 3 days. The populations of T_M cells were analyzed ($n = 4$ biologically independent samples); **h.** $CD8^+$ T_N cells from *Senp1* WT and cKO mice were activated with anti-CD3 (5 μg/mL) and anti-CD28 (2 μg/mL) antibodies + IL-2 for 3 days and then cultured with gradient concentrations of glucose. The populations of T_M cells were analysed ($n = 4$, biologically independent samples); **i-j.** $CD8^+$ T_N cells from *Senp1* WT and cKO mice were activated and then cultured with metformin (2 mM) treatment for 3 days. The populations of T_M cells were analysed ($n = 4$ biologically independent samples, Two-way ANOVA followed by Fisher's LSD test: ***$P < 0.0001$ vehicle vs metformin treatment in *Senp1* WT cells, and $P = 0.058$ vehicle vs metformin treatment in *Senp1* cKO cells). The data are representative of three independent experiments. The summarizing date of were **c, g** and **h** shown in **Fig. 2e, 2h** and **2k** in manuscript. Data are shown as mean ± SEM. n.s. (no significant).

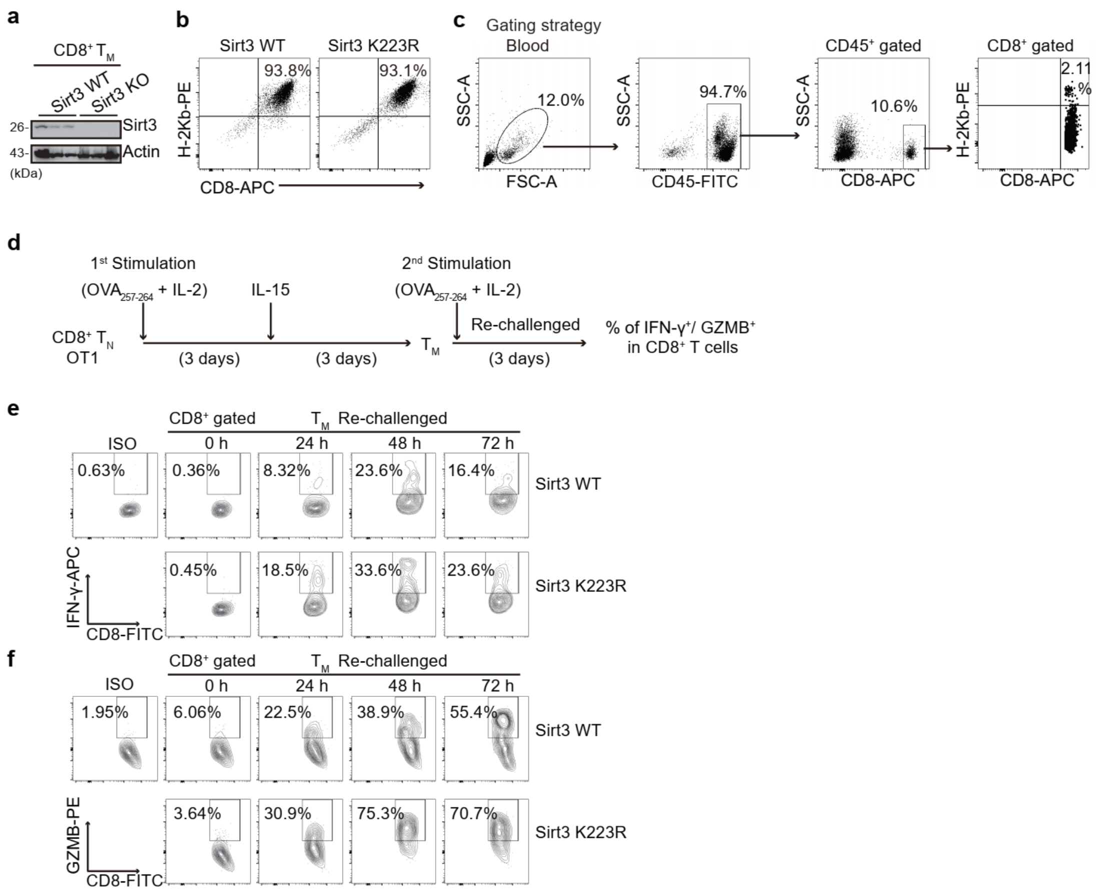

Supplementary Fig. 3 SENP1-Sirt3 axis enhances the survival of T_M cells

a. Sirt3 protein in T_M cells generated from *Sirt3* WT or *Sirt3*-knockout (KO) mice (*n* = 3 mice per group); **b-c.** The percentage of H-2Kb$^+$ in $OVA_{257\text{-}264}$ peptide-activated Sirt3 WT or Sirt3 K223R OT1 CD8$^+$ T cells was detected by FACS **(b)**. The FACS strategy of H-2Kb$^+$ CD8$^+$ T cell populations in recipient blood **(c)**; **d-f.** The analysis of recall antigen responses in T_M cells**.** Experiment design to analyse re-call antigen responses (Re-challenge) in T_M cells **(d)**. The populations of IFN-γ **(e)** or Granzyme B (GZMB) **(f)** positive T cells in T_M cells from Sirt3 WT- or Sirt3 K223R OT1 mice were analyzed by FACS at the indicated time-points after Re-challenged with $OVA_{257\text{-}264}$-peptides *in vitro* (*n* = 5 mice per group). Isotype (ISO). The summarizing date were shown in **Fig. 3l-m** in manuscript.

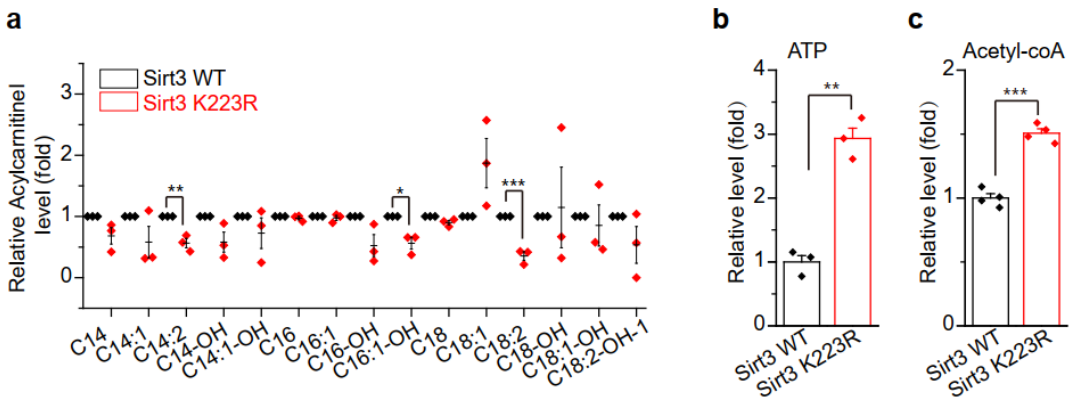

Supplementary Fig. 4 Sirt3 K223R promotes FAO-fuelled OXPHOS in T_M cells.

a-c. Sirt3 K223R T_M cells showed much fewer long-chain acylcarnitine species (*n* = 3 biologically independent samples, Two-tailed unpaired *t* test: $^{**}P = 0.0045$, $^{*}P = 0.010$ and $^{***}P = 0.00074$ for C14:2, C16:1-OH and C18:2 respectively) **(a)**, but more ATP (*n* = 3 biologically independent samples, Two-tailed unpaired *t*-test: $^{**}P = 0.002$) **(b)** and Acetyl-CoA(*n* = 4 biologically independent samples, Two-tailed unpaired *t*-test: $^{***}P < 0.0001$) **(c)** in mitochondria as compared to Sirt3 WT T_M cells. Data are shown as mean ± SEM.

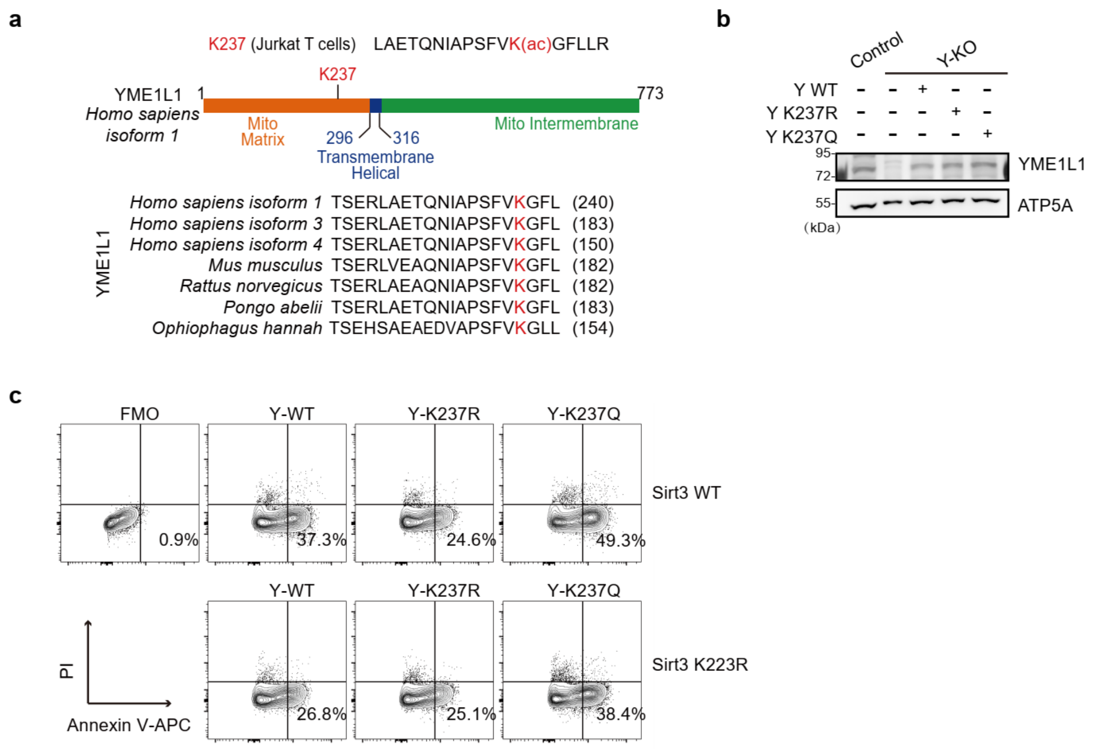

Supplementary Fig. 5 Sirt3 deacetylates YME1L1 and leads to mitochondrial fusion in T_M cells. a. K237 (*Homo sapiens isoform 1*) is a conserved site and locates in mitochondrial matrix domain of YME1l1 protein. acetylation (ac), mitochondrial (Mito); **b.** YME1L1 proteins were blotted with anti-YME1L1 antibody in endogenous-YME1L1 KO HeLa cells infected with Lv-YME1L1-WT (Y-WT), -YME1L1 K237R (Y-K237R), or -YME1L1 K237Q (Y-K237Q); **c.** The T_M cells generated from Sirt3 WT or K223R T_N cells, were infected with lentivirus-YME1L1WT (Lv-Y-WT), -YME1L1-K237Q (Lv-Y-K237R), or YME1L1 K237Q (Lv-Y-K237Q) respectively. The apoptosis of these cells was measured by using Annexin V and Propidium Iodide (PI) staining (*n* = 3 biologically independent samples). The summarizing date were shown in **Fig. 5l, m** manuscript.

Supplementary table 1:

**REAGENT or RESOURCE**	**SOURCE**	**IDENTIFIER**
**Antibodies**		
anti-mouse CD3e	BD Bioscience	Cat#553057; RRID:AB_394590
anti-mouse CD28	BD Bioscience	Cat#553294; RRID:AB_394763
Purified anti-mouse CD16/32	Biolgend	Cat#101302; RRID:AB_312801
APC/Cy7 anti-mouse CD3e	BD Bioscience	Cat#557596; RRID:AB_396759
PerCP/Cy5.5 anti-mouse CD3e	Biolgend	Cat#100327; RRID:AB_893320
FITC anti-mouse CD3e	Ebioscience	Cat#110031-82; RRID:AB_464882
APC anti-mouse CD4	Biolgend	Cat#100412; RRID:AB_312697
APC anti-mouse CD8a	Biolgend	Cat#100712; RRID:AB_312751
Alexa Fluor 594 anti-mouse CD8a	Biolgend	Cat#100758; RRID:AB_2563237
PE anti-mouse CD8a	Biolgend	Cat#100707; RRID:AB_312746
FITC anti-mouse CD8a	Biolgend	Cat#100705; RRID:AB_312744
FITC anti-mouse CD62L	Biolgend	Cat#104406; RRID:AB_313093
PE/Cy7 anti-mouse CD62L	Biolgend	Cat#104418; RRID:AB_313103
APC/Cy7 anti-mouse CD44	Biolgend	Cat#103028; RRID:AB_830785
FITC anti-mouse CD44	BD Bioscience	Cat#561859; RRID:AB_10894581
PE anti-mouse CD25	BD Bioscience	Cat#553075; RRID:AB_394605
PE/Cy7 anti-mouse CD69	Biolgend	Cat#104512; RRID:AB_493564
APC/Cy7 anti-mouse CD127(IL-7Ra)	Biolgend	Cat#135040; RRID:AB_2566161
PE/Cy7 anti-mouse/human KLRG1	Biolgend	Cat#138416; RRID:AB_2561736
PerCP-Cy5.5 anti-Mouse CD45	BD Bioscience	Cat#550994; RRID:AB_394003
FITC anti-mouse CD45.2	Biolegend	Cat#109806; RRID:AB_313443
PerCP/Cy5.5 anti-mouse CD45.2	Biolgend	Cat#109828; RRID:AB_893350
PE anti-mouse IFN-γ	Biolgend	Cat#505808; RRID:AB_315402
APC anti-mouse IFN-γ	Biolgend	Cat#505810; RRID:AB_315404
PE anti-mouse Granzyme B	eBioscience	Cat#12-8898-80; RRID:AB_10853811
FITC anti-mouse CD49d	Biolgend	Cat#103605
Acetylated-Lysine antibody	Cell Signaling Technology	Cat#9441 ; RRID:AB_331805
Anti-Rabbit SUMO1	N/A	(Cai, L. et al. 2017)[3]
OPA1 antibody	Abcam	Cat#ab42364; RRID:AB_944549
YME1L1 antibody	Abcam	Cat#ab170123
OMA1 antibody	Abcam	Cat#ab104316; RRID:AB_10716457
SIRT3 (D22A3) Rabbit mAb	Cell Signaling Technology	Cat#5490; RRID:AB_10828246
SENP1 antibody	Abcam	Cat#108981; RRID:AB_10862449
Recombinant Anti-AMPK alpha 1 (phospho T183) + AMPK alpha 2	Abcam	Cat#ab133448

(phospho T172) antibody [EPR5683]		
Anti-AMPK alpha 1 + AMPK alpha 2 antibody	Abcam	Cat#ab80039; RRID:AB_1603618
Phospho-Acetyl-CoA Carboxylase (Ser79) (D7D11)	CST	Cat#11818S
Anti-Acetyl Coenzyme A Carboxylase antibody	Abcam	Cat#ab45174
LKB1 antibody	CST	Cat#3047S; RRID:AB_2198327
Axin1 antibody	CST	Cat#2087T
Lamtor1 antibody	CST	Cat#8975S; RRID:AB_10860252
Aldolase antibody	Abcam	Cat#ab200049
Anti-Mouse ATP5A	Abcam	Cat#110273; RRID:AB_10858175
Anti-mouse β-Actin (Actin)	Sigma	Cat#A5441; RRID:AB_476744
Anti-Goat Lamin B	Santa Cruz	Cat#sc-6216; RRID:AB_648156
Anti-Rabbit IgG HRP-linked	CST	Cat#7074P2; RRID:AB_2099233
Anti-Mouse IgG HRP-linked	CST	Cat#7076P2; RRID:AB_330924
Anti-Goat IgG HRP-linked	Santa Cruz	Cat#sc-2354; RRID:AB_628490
Purified anti-mouse IFN-γ	Biolegend	Cat#517903
Purified anti-mouse IL-12	Biolegend	Cat#505303
Purified anti-mouse IL-4	Biolegend	Cat#504101
**Chemicals, Peptides, and Recombinant Proteins**		
Recombinant Murine IL-2	PeproTech	Cat#212-12
Recombinant Murine IL-15	PeproTech	Cat#210-15
Recombinant Murine IL-12	PeproTech	Cat#210-12
Recombinant Murine IL-4	PeproTech	Cat#214-14
Recombinant Murine IL-6	PeproTech	Cat#216-16
Recombinant Mouse TGF-beta 1 Protein	R&D System	Cat#7666-MB-005
Ovalbumin (257-264) chicken	Sigma	Cat#S7951
Nicotinamide (NAM)	Selleck	Cat#S1899
Cell Stimulation Cocktail	ebioscience	Cat#00-4975-93
Protein A/G Magnetic Beads	Thermo Scientific	Cat#88802
Metformin	Selleck	Cat#S1950
Compound C	Selleck	Cat#S7306
fructose-1, 6-bisphosphate (FBP)	Santa cruz	Cat#sc-214805A
**Critical Commercial Assays**		
Seahorse XF Cell Mito Stress Test Kit (OCR)	Agilent	Cat#103015-100
Seahorse XF Glycolysis Stress Test Kit(ECAR)	Agilent	Cat#103020-100
Annexin V-FITC Apoptosis	ebioscience	Cat#BMS500FI-100

Detection Kit		
Annexin V-APC Apoptosis Detection Kit	ebioscience	Cat#88-8007-72
Cytofix/Cytoperm Fixation/ Permeabilization Kit	BD Bioscience	Cat#554714
MitoTracker Green	Invitrogen	Cat#M7514
MitoTracker Red	Invitrogen	Cat#M7512
MitoTracker Deep Red	Invitrogen	Cat#M22426
JC-1	Invitrogen	Cat#T3168
CFSE Cell Division Tracker Kit	Biolgend	Cat#423801
T-Select H-2Kb OVA Tetramer -PE	MBL	Cat#TS-5001-1C
7-AAD Viability Staining Solution	Biolgend	Cat#420404
Fixable Viability Dye eFluor 450	eBioscience	Cat#65-0863-14
EasySep Mouse Naive $CD8^+$ T Cell Isolation Kit	STEMCELL	Cat#19858
EasySep Mouse Naive $CD4^+$ T Cell Isolation Kit	STEMCELL	Cat#19765
Fructose-1,6-Bisphosphate Assay Kit	Biovision	Cat#K2036
ATP Colorimetric/Fluorometric Assay Kit	Biovision	Cat#K354-100
Acetyl-CoA Assay Kit	Biovision	Cat#K317-100
**Experimental Models: Cell Lines**		
Human: HEK293T	ATCC	Cat#CRL-3216; RRID:CVCL_0063
Human: HeLa	ATCC	Cat#CCL-2; RRID:CVCL_0030
Human: Jurkat Clone E6-1	ATCC	Cat#TIB-152; RRID:CVCL_0367
OVA-expressing Mouse MC38 colon cancer cells	N/A	(Mender et al., 2020)[4]
**Experimental Models: Organisms/Strains**		
Cd4-Cre mice	The Jackson Laboratory	Cat# JAX:022071, RRID:IMSR_JAX:022071
OT-1 mice	The Jackson Laboratory	Cat# 003831; RRID: IMSR_JAX:003831-UCD
CD45.1 mice	The Jackson Laboratory	Cat# 002014; RRID:IMSR_JAX:002014
Sirt3 knockout (Sirt3 KO) mice	N/A	(Hallows et al., 2011)[5]
Sirt3 K223R mice	N/A	(Wang et al., 2019)[6]
Senp1 $^{flox/flox}$ mice	N/A	(Ferdaoussi et al., 2015)[7]
**Recombinant DNA**		
pcDNA3.1-YME1L1-WT-Flag	This paper	N/A
pcDNA3.1-YME1L1-K237R-Flag	This paper	N/A
pcDNA3.1-YME1L1- K237Q-Flag	This paper	N/A
pCDH-GFP-YME1L1-WT	This paper	N/A

pCDH-GFP-YME1L1- K237R	This paper	N/A
pCDH-GFP-YME1L1- K237Q	This paper	N/A
**Oligonucleotides**		
Human: *YME1L1*gRNA	ggaaccgaccatattacaacagg	
Human: *YME1L1*-Fwd-*Not*I	atatgcggccgcatgttttccttgtcgagcac	
Human: *YME1L1*-Rev-*Not*I	atatgcggccgctatatctcacttccaac	
Human: YME1L1 K237R-Fwd	ccatcattcgtgagggggtttcttttg	
Human: YME1L1 K237R-Rev	caaacgaaaccccctcacgaatgatgg	
Human: YME1L1 K237Q-Fwd	ccatcattcgtgcaggggtttcttttg	
Human: YME1L1 K237Q-Rev	caaacgaaacccctgcacgaatgatgg	
**Software and Algorithms**		
FlowJo v.10.CL	FlowJo	https://www.flowjo.com/
Prism v.7.0e	GraphPad	http://www.graphpad.com
Image-J	NIH	https://imagej.nih.gov/ij/
Image Pro-Plus	Media Cybernetics	http://www.mediacy.com/imagepropl us
LCquan 2.7 software	Thermo Fisher Scientific	https://www.thermofisher.cn
BD FACSuite (1.0.5.3841)	BD Biosciences	www.bdbiosciences.com
BD FACSDiva (6.1.3)	BD Biosciences	www.bdbiosciences.com
RADIUS (2.0)	EMSIS	https://nextcloud.emsis.eu/index.php/ s/b6CrGQ59BzMfkkf
Seahorse Wave (2.6.1.53)	Agilent	www.agilent.com
Leica LAS X Core (3.7.2.22383)	Leica	www.leica-microsystems.com
**Other**		
Seahorse XFe96 Extracellular Flux Analyzer	Agilent Technologies	N/A
BD LSR Fortessa X20	BD Biosciences	N/A
BD FACSVerse^{TM}	BD Biosciences	N/A

Supplementary References:

1 Buck, Michael D. *et al.* Mitochondrial Dynamics Controls T Cell Fate through Metabolic Programming. *Cell* **166**, 63-76, doi:10.1016/j.cell.2016.05.035 (2016).

2 van der Windt, G. J. *et al.* Mitochondrial respiratory capacity is a critical regulator of CD8+ T cell memory development. *Immunity* **36**, 68-78, doi:10.1016/j.immuni.2011.12.007 (2012).

3 Cai, L. *et al.* Proteome-wide Mapping of Endogenous SUMOylation Sites in Mouse Testis. *Mol Cell Proteomics* **16**, 717-727, doi:10.1074/mcp.M116.062125 (2017).

4 Mender, I. *et al.* Telomere Stress Potentiates STING-Dependent Anti-tumor Immunity. *Cancer cell* **38**, 400-411 e406, doi:10.1016/j.ccell.2020.05.020

(2020).
5 Hallows, W. C. *et al.* Sirt3 promotes the urea cycle and fatty acid oxidation during dietary restriction. *Molecular cell* **41**, 139-149, doi:10.1016/j.molcel.2011.01.002 (2011).
6 Wang, T. *et al.* SENP1-Sirt3 Signaling Controls Mitochondrial Protein Acetylation and Metabolism. *Molecular cell* **75**, 823-834., doi:10.1016/j.molcel.2019.06.008 (2019).
7 Ferdaoussi, M. *et al.* Isocitrate-to-SENP1 signaling amplifies insulin secretion and rescues dysfunctional beta cells. *The Journal of clinical investigation* **125**, 3847-3860, doi:10.1172/JCI82498 (2015).
