# Supplementary material for: Glucose limitation activates AMPK coupled SENP1-Sirt3 signalling in mitochondria for T cell memory development
Source: Nat Commun. 2021 Jul 16;12:4371. doi: 10.1038/s41467-021-24619-2 (PMC8285428; doi:10.1038/s41467-021-24619-2)
Supplement: Supplementary file 2 — Reporting Summary [file 41467_2021_24619_MOESM2_ESM.pdf]

nature research

Corresponding author(s): Jinke Cheng; Tianshi Wang; Qi Wang

Last updated by author(s): Jun 7, 2021

Reporting Summary

Nature Research wishes to improve the reproducibility of the work that we publish. This form provides structure for consistency and transparency in reporting. For further information on Nature Research policies, see our Editorial Policies and the Editorial Policy Checklist.

Statistics

For all statistical analyses, confirm that the following items are present in the figure legend, table legend, main text, or Methods section.

n/a	Confirmed	
☐	☒	The exact sample size (n) for each experimental group/condition, given as a discrete number and unit of measurement
☐	☒	A statement on whether measurements were taken from distinct samples or whether the same sample was measured repeatedly
☐	☒	The statistical test(s) used AND whether they are one- or two-sided<br>*Only common tests should be described solely by name; describe more complex techniques in the Methods section.*
☐	☒	A description of all covariates tested
☐	☒	A description of any assumptions or corrections, such as tests of normality and adjustment for multiple comparisons
☐	☒	A full description of the statistical parameters including central tendency (e.g. means) or other basic estimates (e.g. regression coefficient) AND variation (e.g. standard deviation) or associated estimates of uncertainty (e.g. confidence intervals)
☐	☒	For null hypothesis testing, the test statistic (e.g. F, t, r) with confidence intervals, effect sizes, degrees of freedom and P value noted<br>*Give P values as exact values whenever suitable.*
☒	☐	For Bayesian analysis, information on the choice of priors and Markov chain Monte Carlo settings
☒	☐	For hierarchical and complex designs, identification of the appropriate level for tests and full reporting of outcomes
☒	☐	Estimates of effect sizes (e.g. Cohen's d, Pearson's r), indicating how they were calculated

Our web collection on statistics for biologists contains articles on many of the points above.

Software and code

Policy information about availability of computer code

Data collection	LCquan 2.7 software (Thermofisher Scientific) for metabolomics data collection;<br>BD FACSVerseTM and BD FACSuite (1.0.5.3841)software or LSR Fortessa X-20 cell analyzer (BD Biosciences) and BD FACSDiva (6.1.3) software for flow cytometry data collection;<br>HITACHI H-7650 or PHILIPS CM-120 and RADIUS, 2.0 (EMSIS GmbH, Muenster, Germany) software for transmission electron microscope data collection;<br>96 well XF Extracellular Flux Analyzer (EFA) (Seahorse Bioscience) and Seahorse Wave (2.6.1.53) software for data collection;<br>Leica TCS Sp8 STED and Leica LAS X Core (3.7.2.22383) software for confocal fluorescence microscopy data collection.
Data analysis	ImageJ (1.52a), Image-Pro plus (version 6.0) software and Adobe Photoshop CS6 to process image data;<br>Adobe Illustrator CS6 to assemble figures;<br>Graphpad Prism 7.0a to perform statistical analyses;<br>FlowJo software (v.10.CL) to do flow cytometry analyses.

For manuscripts utilizing custom algorithms or software that are central to the research but not yet described in published literature, software must be made available to editors and reviewers. We strongly encourage code deposition in a community repository (e.g. GitHub). See the Nature Research guidelines for submitting code & software for further information.

Data

Policy information about availability of data

All manuscripts must include a data availability statement. This statement should provide the following information, where applicable:

- Accession codes, unique identifiers, or web links for publicly available datasets
- A list of figures that have associated raw data
- A description of any restrictions on data availability

All the data of this study are available.

Field-specific reporting

Please select the one below that is the best fit for your research. If you are not sure, read the appropriate sections before making your selection.

[x] Life sciences [ ] Behavioural & social sciences [ ] Ecological, evolutionary & environmental sciences

For a reference copy of the document with all sections, see nature.com/documents/nr-reporting-summary-flat.pdf

Life sciences study design

All studies must disclose on these points even when the disclosure is negative.

Sample size	Sample size (or number of repeats) was chosen based on what is common in the field, and what was practical to do.
Data exclusions	No data were excluded from the analyses.
Replication	All experiments were replicated independently. Number of repeats is provided in the text and figure legends where appropriate.
Randomization	All experiments were randomly distributed and assigned to different treatment groups prior to the start of the treatment. Sample randomization is not applicable to the other experiments presented here.
Blinding	Investigators were blinded during data collection and/or analysis.

Reporting for specific materials, systems and methods

We require information from authors about some types of materials, experimental systems and methods used in many studies. Here, indicate whether each material, system or method listed is relevant to your study. If you are not sure if a list item applies to your research, read the appropriate section before selecting a response.

Materials & experimental systems

n/a	Involved in the study	
[ ]	[x]	Antibodies
[ ]	[x]	Eukaryotic cell lines
[x]	[ ]	Palaeontology and archaeology
[ ]	[x]	Animals and other organisms
[x]	[ ]	Human research participants
[x]	[ ]	Clinical data
[x]	[ ]	Dual use research of concern

Methods

n/a	Involved in the study	
[x]	[ ]	ChIP-seq
[ ]	[x]	Flow cytometry
[x]	[ ]	MRI-based neuroimaging

Antibodies

Antibodies used

1. anti-mouse CD3e BD Bioscience Cat#553057; RRID:AB_394590 (1:1000 dilution)
2. anti-mouse CD28 BD Bioscience Cat#553294; RRID:AB_394763 (1:1000 dilution)
3. Purified anti-mouse CD16/32 Biolgend Cat#101302; RRID:AB_312801 (1:1000 dilution)
4. APC/Cy7 anti-mouse CD3e BD Bioscience Cat#557596; RRID:AB_396759 (1:1000 dilution)
5. PerCP/Cy5.5 anti-mouse CD3e Biolgend Cat#100327; RRID:AB_893320 (1:1000 dilution)
6. FITC anti-mouse CD3e Ebioscience Cat#110031-82; RRID:AB_ 464882 (1:1000 dilution)
7. APC anti-mouse CD4 Biolgend Cat#100412; RRID:AB_312697 (1:1000 dilution)
8. APC anti-mouse CD8a Biolgend Cat#100712; RRID:AB_312751 (1:1000 dilution)
9. Alexa Fluor 594 anti-mouse CD8a Biolgend Cat#100758; RRID:AB_2563237 (1:1000 dilution)
10. PE anti-mouse CD8a Biolgend Cat#100707; RRID:AB_312746 (1:1000 dilution)
11. FITC anti-mouse CD8a Biolgend Cat#100705; RRID:AB_312744 (1:1000 dilution)
12. FITC anti-mouse CD62L Biolgend Cat#104406; RRID:AB_313093 (1:1000 dilution)

13. PE/Cy7 anti-mouse CD62L Biolgend Cat#104418; RRID:AB_313103 (1:1000 dilution)
14. APC/Cy7 anti-mouse CD44 Biolgend Cat#103028; RRID:AB_830785 (1:1000 dilution)
15. FITC anti-mouse CD44 BD Bioscience Cat#561859; RRID:AB_10894581 (1:1000 dilution)
16. PE anti-mouse CD25 BD Bioscience Cat#553075; RRID:AB_394605 (1:1000 dilution)
17. PE/Cy7 anti-mouse CD69 Biolgend Cat#104512; RRID:AB_ 493564 (1:1000 dilution)
18. APC/Cy7 anti-mouse CD127(1L-7Ra) Biolgend Cat#135040; RRID:AB_2566161 (1:1000 dilution)
19. PE/Cy7 anti-mouse/human KLRGl Biolgend Cat#138416; RRID:AB_2561736 (1:1000 dilution)
20. PerCP-CyS.5 anti-Mouse CD45 BD Bioscience Cat#550994; RRID:AB_394003 (1:1000 dilution)
21. FITC anti-mouse CD45.2 Biolegend Cat#109806; RRID:AB_313443 (1:1000 dilution)
22. PerCP/Cy5.5 anti-mouse CD45.2 Biolgend Cat#109828; RRID:AB_893350 (1:1000 dilution)
23. PE anti-mouse IFN-y Biolgend Cat#505808; RRID:AB_315402 (1:1000 dilution)
24. APC anti-mouse IFN-y Biolgend Cat#505810; RRID:AB_315404 (1:1000 dilution)
25. PE anti-mouse Granzyme B eBioscience Cat#l2-8898-80; RRID:AB_l0853811 (1:1000 dilution)
26. FITC anti-mouse CD49d Biolgend Cat#103605 (1:1000 dilution)
27. Acetylated-Lysine antibody CST Cat#9441; RRID:AB_331805 (1:300 dilution for IP; 1:1000 dilution for WB)
28. Anti-Rabbit SUMO1 (Cai et al., 2017) (1:300 dilution for IP; 1:1000 dilution for WB)
29. OPA1 antibody Abcam Cat#ab42364; RRID:AB_944549 (1:1000 dilution)
30. YME1L1 antibody Abcam Cat#ab170123 (1:1000 dilution)
31. OMA1 antibody Abcam Cat#ab154949; RRID:AB_10716457 (1:1000 dilution)
32. SIRT3 (D22A3) Rabbit mAb CST Cat#5490; RRID:AB_10828246 (1:3000 dilution)
33. SENP1 antibody Abcam Cat#108981; RRID:AB_10862449 (1:3000 dilution)
34. Recombinant Anti-AMPK alpha 1 (phospho T183) + AMPK alpha 2 (phospho T172) antibody [EPR5683] Abcam Cat#ab133448 (1:1000 dilution)
35. Anti-AMPK alpha 1 + AMPK alpha 2 antibody Abcam Cat#ab80039; RRID:AB_1603618 (1:1000 dilution)
36. Phospho-Acetyl-CoA Carboxylase (Ser79) (D7D11) CST Cat#11818 (1:1000 dilution)
37. Anti-Acetyl Coenzyme A Carboxylase antibody Abcam Cat#ab45174 (1:1000 dilution)
38. LKB1 antibody CST Cat#3047; RRID:AB_2198327 (1:2000 dilution)
39. Axin1 antibody CST Cat#2087 (1:1000 dilution)
40. Lamtorl antibody CST Cat#8975; RRID:AB_l0860252 (1:1000 dilution)
41. Aldolase antibody Abcam Cat#ab200049 (1:1000 dilution)
42. Anti-Mouse ATP5A Abcam Cat#110273; RRID:AB_l0858175 (1:5000 dilution)
43. Anti-mouse beta-Actin Sigma Cat#A5441; RRID:AB_ 476744 (1:5000 dilution)
44. Anti-Goat Lamin B Santa Cruz Cat#sc-374015; RRID:AB_648156 (1:5000 dilution)
45. Anti-Rabbit lgG HRP-linked CST Cat#7074; RRID:AB_2099233 (1:3000 dilution)
46. Anti-Mouse lgG HRP-linked CST Cat#7076; RRID:AB_330924 (1:3000 dilution)
47. Anti-Goat lgG HRP-linked Santa Cruz Cat#sc-2354; RRID:AB_628490 (1:3000 dilution)

Validation

The antibodies used for flow cytometry and western blot are widely used and well validated in the literature.
1. https://www.bdbiosciences.com/cn/applications/research/t-cell-immunology/th-1-cells/surface-markers/mouse/purified-nale-hamster-anti-mouse-cd3e-145-2c11/p/553057
2. https://www.bdbiosciences.com/cn/applications/research/t-cell-immunology/regulatory-t-cells/surface-markers/mouse/purified-nale-hamster-anti-mouse-cd28-3751/p/553294
3. https://www.biolegend.com/en-us/search-results/purified-anti-mouse-cd16-32-antibody-190
4. https://www.bdbiosciences.com/cn/applications/research/t-cell-immunology/th-1-cells/surface-markers/mouse/apc-cy7-hamster-anti-mouse-cd3e-145-2c11/p/557596
5. https://www.biolegend.com/en-us/products/percp-cyanine5-5-anti-mouse-cd3epsilon-antibody-4191
6. https://www.thermofisher.cn/cn/zh/antibody/product/CD3e-Antibody-clone-145-2C11-Monoclonal/11-0031-82
7. https://www.biolegend.com/en-us/products/apc-anti-mouse-cd4-antibody-245
8. https://www.biolegend.com/en-us/products/apc-anti-mouse-cd8a-antibody-150
9. https://www.biolegend.com/en-us/products/alexa-fluor-594-anti-mouse-cd8a-antibody-9608
10. https://www.biolegend.com/en-us/products/pe-anti-mouse-cd8a-antibody-155
11. https://www.biolegend.com/en-us/products/fitc-anti-mouse-cd8a-antibody-153
12. https://www.biolegend.com/en-us/products/fitc-anti-mouse-cd62l-antibody-384
13. https://www.biolegend.com/en-us/products/apc-cyanine7-anti-mouse-human-cd44-antibody-3933
14. https://www.biolegend.com/en-us/products/apc-cyanine7-anti-mouse-human-cd44-antibody-3933
15. https://www.bdbiosciences.com/cn/applications/research/t-cell-immunology/t-follicular-helper-tfh-cells/surface-markers/mouse/fitc-rat-anti-mouse-cd44-im7/p/561859
16. https://www.bdbiosciences.com/cn/applications/research/t-cell-immunology/regulatory-t-cells/surface-markers/mouse/pe-rat-anti-mouse-cd25-3c7/p/553075
17. https://www.biolegend.com/en-us/products/pe-cyanine7-anti-mouse-cd69-antibody-3168
18. https://www.biolegend.com/en-us/products/apc-cyanine7-anti-mouse-cd127-il-7ralpha-antibody-12267
19. https://www.biolegend.com/en-us/products/pe-cyanine7-anti-mouse-human-klrg1-mafa-antibody-8312
20. https://www.bdbiosciences.com/cn/applications/research/stem-cell-research/cancer-research/mouse/percp-cy55-rat-anti-mouse-cd45-30-f11/p/550994
21. https://www.biolegend.com/en-us/products/fitc-anti-mouse-cd45-2-antibody-6
22. https://www.biolegend.com/en-us/products/percp-cyanine5-5-anti-mouse-cd452-antibody-4271
23. https://www.biolegend.com/en-us/products/pe-anti-mouse-ifn-gamma-antibody-997
24. https://www.biolegend.com/en-us/products/apc-anti-mouse-ifn-gamma-antibody-993

25. https://www.thermofisher.cn/cn/zh/antibody/product/Granzyme-B-Antibody-clone-NGZB-Monoclonal/12-8898-80
26. https://www.biolegend.com/en-us/search-results/fitc-anti-mouse-cd49d-antibody-438
27. https://www.cellsignal.cn/products/primary-antibodies/acetylated-lysine-antibody/9441?site-search-type=Products&N=4294956287&Ntt=9441&fromPage=plp&_requestid=1050925
28. Vailidated in Cai L, Tu J, Song L, et al. Proteome-wide Mapping of Endogenous SUMOylation Sites in Mouse Testis. Mol Cell Proteomics. 2017;16(5):717-727. doi:10.1074/mcp.M116.062125
29. https://www.abcam.com/opa1-antibody-ab42364.html
30. https://www.abcam.com/yme1l1-antibody-ab170123.html
31. https://www.abcam.com/oma1-antibody-ab154949.html
32. https://www.cellsignal.cn/products/primary-antibodies/sirt3-d22a3-rabbit-mab/5490?site-search-type=Products&N=4294956287&Ntt=5490&fromPage=plp&_requestid=1051774
33. https://www.abcam.com/senp1-antibody-epr3844-ab108981.html
34. https://www.abcam.com/ampk-alpha-1-phospho-t183--ampk-alpha-2-phospho-t172-antibody-epr5683-ab133448.html
35. https://www.abcam.com/ampk-alpha-1-ampk-alpha-2-antibody-342-ab80039.html
36. https://www.cellsignal.cn/products/primary-antibodies/phospho-acetyl-coa-carboxylase-ser79-d7d11-rabbit-mab/11818?site-search-type=Products&N=4294956287&Ntt=phospho-acetyl-coa+carboxylase+%28ser79%29&fromPage=plp
37. https://www.abcam.com/acetyl-coenzyme-a-carboxylase-antibody-ep687y-ab45174.html
38. https://www.cellsignal.cn/products/primary-antibodies/lkb1-d60c5-rabbit-mab/3047?_=1622348289653&Ntt=3047%20lkb1&tahead=true
39. https://www.cellsignal.cn/products/primary-antibodies/axin1-c76h11-rabbit-mab/2087?site-search-type=Products&N=4294956287&Ntt=2087t&fromPage=plp&_requestid=1052400
40. https://www.cellsignal.cn/products/primary-antibodies/lamtor1-c11orf59-d11h6-xp-rabbit-mab/8975?site-search-type=Products&N=4294956287&Ntt=8975&fromPage=plp&_requestid=1052477
41. https://www.abcam.com/aldolase--aldolase-c-antibody-epr19355-ab200049.html
42. https://www.abcam.com/atp5a-antibody-7h10bd4f9-ab110273.html
43. https://www.sigmaaldrich.cn/CN/zh/product/sigma/a5441?context=product
44. https://www.scbt.com/p/lamin-b1-antibody-b-10
45. https://www.cellsignal.cn/products/secondary-antibodies/anti-rabbit-igg-hrp-linked-antibody/7074?site-search-type=Products&N=4294956287&Ntt=7074p2&fromPage=plp&_requestid=1053081
46. https://www.cellsignal.cn/products/secondary-antibodies/anti-mouse-igg-hrp-linked-antibody/7076?site-search-type=Products&N=4294956287&Ntt=7076p2&fromPage=plp&_requestid=1053009
47. https://www.scbt.com/p/mouse-anti-goat-igg-hrp?requestFrom=search

Eukaryotic cell lines

Policy information about cell lines

Cell line source(s)

Human: HEK293T ATCC Cat#CRL-3216; RRID:CVCL_0063
Human: HeLa ATCC Cat#CCL-2; RRID:CVCL_0030
Human: Jurkat Clone E6-1 ATCC Cat#TIB-152; RRID:CVCL_0367
OVA-expressing Mouse MC38 colon cancer cells (MC38-OVA), were originally made by Yang-Xin Fu Lab (UT South western Medical Center, USA), and were obtained from Yang-Xin Fu Lab and Shanghai Institute of Immunology)

Authentication

The cell lines were not authenticated. All cell lines were kept at low passages in order to maintain their identity.

Mycoplasma contamination

All cell lines were tested negative for mycoplasma contamination.

Commonly misidentified lines
(See ICLAC register)

No commonly misidentified cell lines were used in this work.

Animals and other organisms

Policy information about studies involving animals; ARRIVE guidelines recommended for reporting animal research

Laboratory animals

C57BL/6 Sirt3 wild type and K223R mice were described in our previous studies (Wang et al., 2019); C57BL/6 CD45.1 mice (the Jackson Laboratory Cat# 002014; RRID:IMSR_JAX:002014), CD4-Cre mice (the Jackson Laboratory Cat# JAX:022071, RRID:IMSR_JAX:022071) and major histocompatibility complex (MHC) class I-restricted OVA specific TCR OT1 transgenic mice (the Jackson Laboratory Cat# 003831; RRID: IMSR_JAX:003831-UCD) were obtained from Shanghai Institute of Immunology; Sirt3 knockout (Sirt3 KO) mice (Hallows et al., 2011) were obtained from Prof. Shimin Zhao's Laboratory (Fudan University, China); Sirt3 WT or K223R mice were crossed to OT1 transgenic mice to generate Sirt3 WT or K223R OT1 mice; Senp1 flox/flox mice (Ferdaoussi et al., 2015) were crossed with CD4-Cre mice to create T cell- specific SENP1 cKO mice (Senp1 flox/flox x CD4-Cre).
All mice were bred and maintained under a 12-h reverse light/dark cycle and specific pathogen free (SPF) conditions. The animal facility was maintained at a temperature of 22 ± 2 °C with 40-70 % humidity. Age- and sex-matched male and female adult (6-8 week-old) mice were used in each independent experiment.

Wild animals

No wild animals were employed in the study.

Field-collected samples

No field-collected samples were used in this study.

Ethics oversight	The animal experiments were performed in strict accordance with the 'Guide for the Care and Use of Laboratory Animals ', which were approved by the Experimental Animal Ethical Committee at Shanghai Jiao Tong University School of Medicine.

Note that full information on the approval of the study protocol must also be provided in the manuscript.

Flow Cytometry

Plots

Confirm that:

- [x] The axis labels state the marker and fluorochrome used (e.g. CD4-FITC).
- [x] The axis scales are clearly visible. Include numbers along axes only for bottom left plot of group (a 'group' is an analysis of identical markers).
- [x] All plots are contour plots with outliers or pseudocolor plots.
- [x] A numerical value for number of cells or percentage (with statistics) is provided.

Methodology

Sample preparation	Fluorochrome-labelled antibodies (Biolegend, eBioscience and BD Pharmingen), MitoTracker (Green/Red), or JC-1 (Invitrogen) staining were performed according to the manufacturer's instructions; OVA-specific CD8+ T cells from blood or MC38-OVA tumor were staining with H2-Kb OVA257-264 MHC-peptide tetramers (MBL, Japan); CD8+naive T cells were sorted using a MoFlo Astrios (BeckMan) or isolated by EasySep Mouse Naive CD8+ T cell Isolation Kit (STEMCELL).
Instrument	Cells were collected on BD FACSVerseTM or LSR Fortessa X-20 cell analyzer (BD Biosciences).
Software	Data were analyzed using FlowJo (TreeStar) software.
Cell population abundance	The purities of the sorted CD8+ naive T cells were 95-99%.
Gating strategy	CD8+ naive T cells (CD8+CD44-CD62L+), CD8+ central memory T cells (CD8+CD44+CD62L+).

- [x] Tick this box to confirm that a figure exemplifying the gating strategy is provided in the Supplementary Information.
